# Clinical and Radiographic Outcomes of Calcium-Silicate-Based Materials in Primary-Tooth Pulpotomy: An Umbrella Review

**DOI:** 10.3390/jcm15166379

**Published:** 2026-08-18

**Authors:** Carlos M. Ardila, Anny M. Vivares-Builes, Eliana Pineda-Vélez

**Affiliations:** 1 Department of Periodontics, Saveetha Institute of Medical and Technical Sciences, Saveetha Dental College and Hospitals, Saveetha University, Chennai 600077, India; 2 Biomedical Stomatology Research Group, Basic Sciences Department, Faculty of Dentistry, Universidad de Antioquia U de A, Medellín 050010, Colombia; anny.vivares@uam.edu.co (A.M.V.-B.); eliana.pineda@uam.edu.co (E.P.-V.); 3 Faculty of Dentistry, Institución Universitaria Visión de las Américas, Medellín 050040, Colombia

**Keywords:** calcium silicate, mineral trioxide aggregate, pulpotomy, tooth, deciduous

## Abstract

**Background/Objectives:** Calcium-silicate-based materials, many of which are commonly classified as bioceramics, are increasingly used for pulpotomy in vital primary teeth. This umbrella review synthesized systematic reviews and meta-analyses evaluating their clinical and radiographic outcomes. **Methods:** Eligible reports were systematic reviews of human primary-tooth pulpotomy involving calcium-silicate-based materials and reporting clinical and/or radiographic outcomes. MEDLINE/PubMed, Embase, and Scopus were searched from inception through June 2026. Methodological quality was appraised using AMSTAR-2, overlap was quantified using the Corrected Covered Area (CCA), and certainty was assessed using a GRADE-informed approach. Findings were integrated through structured narrative synthesis and review-level functional meta-synthesis. The protocol was registered in PROSPERO (CRD420261338396). **Results:** Eleven systematic reviews were included, comprising 78 unique primary-study reports and 180 study occurrences; aggregate participant totals were not calculated because of primary-study overlap. AMSTAR-2 confidence ranged from critically low to moderate, and overlap was high (CCA 13.1%). MTA and Biodentine showed broadly comparable clinical and radiographic outcomes through 24 months. Evidence for Biodentine versus formocresol was less consistent: one meta-analysis reported more favorable radiographic outcomes with Biodentine; whereas, another found no substantial difference; clinical superiority was not consistently demonstrated. Comparisons with ferric sulfate were generally inconclusive, and evidence for newer calcium-silicate-based formulations remained limited. No material demonstrated sustained long-term superiority. Certainty was predominantly low, with very low certainty for several sparsely studied comparisons. **Conclusions:** Contemporary calcium-silicate-based materials show high clinical and radiographic success in vital primary-tooth pulpotomy, but comparative conclusions remain constrained by methodological limitations, high overlap, and limited certainty.

## 1. Introduction

Preserving the function and integrity of primary teeth until their natural exfoliation remains a fundamental objective of pediatric dentistry. Premature loss of primary molars has been associated with loss of arch length, malocclusion, altered mastication, and speech disturbances, which may ultimately compromise the development of the permanent dentition [1]. Consequently, therapeutic strategies aimed at maintaining pulpal vitality in cariously exposed primary teeth have received considerable attention in clinical practice and research.

Among vital pulp therapy procedures, pulpotomy is widely considered the treatment of choice when the coronal pulp is inflamed but the radicular pulp remains vital [2]. The procedure consists of removing the infected or inflamed coronal pulp tissue followed by placement of a biomaterial over the remaining radicular pulp to promote healing and maintain vitality. The biological success of pulpotomy therefore depends largely on the properties of the material used to protect and stimulate the remaining pulp tissue.

Historically, several materials have been used as pulpotomy medicaments, including formocresol, ferric sulfate, zinc oxide–eugenol, and calcium hydroxide [3]. Although these materials demonstrated acceptable clinical outcomes, concerns have been raised regarding their biological compatibility and long-term safety. In particular, formocresol has been questioned because of the potential cytotoxic and mutagenic effects associated with formaldehyde-containing compounds [4]. These concerns have stimulated extensive research aimed at identifying alternative biomaterials capable of improving biological outcomes while maintaining high clinical success rates.

In recent decades, calcium-silicate-based materials, including mineral trioxide aggregate (MTA), Biodentine, and related bioactive cements, have emerged as promising alternatives for pulpotomy procedures. In this review, “calcium-silicate-based materials” is used as the principal collective descriptor for these materials, many of which are also commonly classified as bioceramic materials. The term “bioceramic” is retained when referring to the terminology or specific material classifications used in the original studies and systematic reviews. Calcium-silicate-based materials exhibit several biological advantages, including excellent sealing ability, high biocompatibility, antimicrobial activity, and the ability to promote pulp healing and reparative hard-tissue formation [5]. As a result, they have become increasingly adopted in pediatric endodontics and are considered among the biologically favorable options for vital pulp therapy in primary teeth.

Randomized clinical trials have evaluated the clinical and radiographic outcomes of pulpotomy performed with calcium-silicate-based materials in primary teeth, generally reporting favorable treatment outcomes [6]. However, individual clinical trials often include relatively small sample sizes and heterogeneous methodologies, making it difficult to draw definitive conclusions regarding the comparative effectiveness of different calcium-silicate-based materials.

To address these limitations, several systematic reviews and meta-analyses have been conducted in recent years. Previous evidence syntheses have reported that mineral MTA demonstrated high clinical success rates and favorable outcomes compared with conventional pulpotomy agents; whereas, Biodentine and MTA showed comparable clinical and radiographic success rates in primary molar pulpotomy. These reviews also suggested that bioactive materials may represent effective alternatives in pediatric vital pulp therapy [7,8,9].

More recent systematic reviews have expanded the scope of this evidence. Evidence syntheses evaluating calcium-silicate-based materials, including formulations classified as first- and second-generation bioceramics, have reported high clinical and radiographic success rates across randomized clinical trials, with no clear superiority of newer formulations over established materials [10,11]. In addition, recent clinical trials published between 2023 and 2025 have continued to demonstrate high clinical and radiographic success rates when MTA and related calcium-silicate-based formulations in pulpotomy procedures [12,13].

Although several systematic reviews and meta-analyses have evaluated calcium-silicate-based materials for primary-tooth pulpotomy, they address different material comparisons, outcomes, and follow-up intervals and do not provide entirely concordant comparative conclusions. Some reviews report no difference between MTA and Biodentine; whereas, others identify time-specific radiographic differences or more favorable radiographic outcomes for Biodentine compared with formocresol [7,8,9,10,11]. Moreover, these reviews frequently synthesize many of the same primary trials, creating potential redundancy and an inflated impression of independent confirmation. No previous overview has simultaneously examined these review-level discrepancies, quantified primary-study overlap, appraised methodological confidence, and evaluated the certainty of the comparative evidence. The rationale for the present umbrella review was therefore not to reproduce existing efficacy estimates, but to determine how independent, consistent, and methodologically reliable the available review-level evidence actually is.

Therefore, the aim of the present umbrella review was to evaluate the clinical and radiographic success of pulpotomy procedures performed with calcium-silicate-based materials in vital primary teeth by synthesizing evidence from existing systematic reviews and meta-analyses. In addition, this study aimed to assess the methodological quality of the included systematic reviews, quantify the degree of overlap among their primary studies, evaluate the certainty of the evidence, and integrate review-level findings according to material comparison, outcome type, and follow-up duration.

## 2. Materials and Methods

### 2.1. Study Design and Protocol Registration

This study was conducted as an umbrella review (overview of systematic reviews) designed to synthesize evidence from systematic reviews and meta-analyses evaluating the clinical and radiographic success of pulpotomy procedures performed with calcium-silicate-based materials in vital primary teeth. Umbrella reviews provide a higher-level synthesis of evidence by integrating results from multiple systematic reviews addressing related clinical questions while allowing assessment of redundancy and overlap among primary studies [14,15]. The reporting of this umbrella review was guided by the Preferred Reporting Items for Overviews of Reviews (PRIOR) statement, an overview-specific reporting guideline developed for overviews of reviews of healthcare interventions [16]. PRISMA 2020 was additionally used as complementary guidance for general systematic-review reporting elements and presentation of the study-selection process [17]. A completed PRIOR checklist is provided as a Supplementary File S1.

The protocol for this umbrella review was prospectively registered in the International Prospective Register of Systematic Reviews (PROSPERO; registration number CRD420261338396) database. The methodological approach was informed by guidance from the Joanna Briggs Institute (JBI) Manual for Evidence Synthesis for umbrella reviews and by established methodological recommendations for conducting overviews of systematic reviews [18].

### 2.2. Eligibility Criteria

Eligibility criteria were defined a priori according to the Population, Intervention, Comparator, Outcomes, and Study Design (PICOS) framework adapted for umbrella reviews. Accordingly, the PICO question guiding this umbrella review was: “In vital primary teeth undergoing pulpotomy, how do calcium-silicate-based materials compare with conventional pulpotomy medicaments or other calcium-silicate-based materials in terms of clinical and radiographic success?”

Population: Eligible systematic reviews evaluated pulpotomy procedures performed in vital primary teeth or primary molars. Reviews focusing exclusively on permanent teeth were excluded. Reviews including both primary and permanent teeth or mixed dentitions were eligible only when the findings for primary teeth were reported separately.

Intervention: Eligible interventions included pulpotomy procedures performed using calcium-silicate-based materials, including mineral trioxide aggregate (MTA), Biodentine, NeoMTA, and other bioactive calcium-silicate cements.

Comparator: Eligible comparisons included conventional pulpotomy medicaments, such as formocresol, ferric sulfate, and calcium hydroxide, as well as comparisons between different calcium-silicate-based materials.

Outcomes: Reviews were required to report at least one relevant clinical or radiographic outcome following pulpotomy. The primary outcomes were clinical success and radiographic success. Additional outcomes of interest included treatment failure, adverse events, and the need for retreatment.

Study design: Systematic reviews, with or without meta-analysis, that synthesized human clinical intervention studies were eligible. The primary studies included in these reviews could consist of randomized or non-randomized clinical intervention studies.

Narrative reviews, scoping reviews, editorials, commentaries, and other non-systematic literature summaries were excluded. Systematic reviews were also excluded when they did not evaluate an eligible bioceramic or calcium-silicate material, did not report outcomes specifically for primary teeth, or did not provide clinical or radiographic pulpotomy outcomes relevant to the objectives of this umbrella review.

Eligibility was applied at the systematic-review level. A review was considered eligible when its review question or a prespecified, distinct review-level synthesis addressed primary-tooth pulpotomy, included at least one calcium-silicate-based material as an intervention or comparator, preserved an eligible comparison group, and reported relevant clinical and/or radiographic outcomes. For reviews with a broader scope, the mere presence of individual eligible primary studies was not considered sufficient when the relevant evidence was not organized as a distinct material-centered primary-tooth pulpotomy synthesis. Reviews whose relevant evidence lacked an eligible comparator group or outcome data were excluded.

### 2.3. Information Sources and Search Strategy

A comprehensive literature search was conducted in MEDLINE via PubMed, Embase, and Scopus from database inception through June 2026. The final search update was completed in June 2026. No restrictions were applied regarding publication year or language. The literature searches were performed independently by AMVB and EPV.

The search strategy was structured around four main conceptual blocks: (1) pulpotomy or vital pulp therapy; (2) primary, deciduous, or primary molar teeth; (3) calcium-silicate-based materials and related terminology, including “bioceramic,” “calcium silicate,” mineral trioxide aggregate, and Biodentine; and (4) systematic reviews or meta-analyses. Controlled vocabulary terms and free-text keywords were combined according to the indexing characteristics of each database. Synonymous terms within each conceptual block were combined using the Boolean operator OR; whereas, the four conceptual blocks were combined using AND.

In PubMed, the strategy used Title/Abstract fields and the systematic review publication type. In Scopus, terms were searched within the title, abstract, and keyword fields using the TITLE-ABS-KEY function. In Embase, Emtree terms were combined with title and abstract keywords. The complete database-specific search strings are provided in Supplementary Table S1.

Additional searches included screening the reference lists of the included systematic reviews and performing forward citation searches of relevant publications. When clarification of methodological information was required, the corresponding authors of the reviews were contacted.

### 2.4. Study Selection

All records identified through the database search were imported into reference management software, and duplicate records were removed prior to screening. The selection process was conducted in two stages. First, titles and abstracts were independently screened by AMVB and EPV to identify potentially relevant studies. Second, the full texts of all potentially eligible articles were retrieved and independently assessed by the same reviewers. Any disagreements during the screening process were resolved through discussion and consensus. When consensus could not be reached, CMA acted as the third reviewer and made the final eligibility decision.

### 2.5. Data Extraction

Data extraction was performed independently by AMVB and EPV using a standardized data extraction form specifically developed for this umbrella review. Any discrepancies were resolved through discussion and consensus, with CMA consulted when necessary. Extracted information included the first author and year of publication of each systematic review, the number and type of primary studies included, the number of randomized clinical trials, the types of pulpotomy materials evaluated, comparator interventions, reported outcomes, pooled effect estimates when meta-analyses were conducted, and the comparison-specific conclusions of each review.

### 2.6. Methodological Quality Assessment

The methodological quality of the included systematic reviews was assessed using AMSTAR-2 (A Measurement Tool to Assess Systematic Reviews 2), a validated instrument designed to critically appraise systematic reviews that include randomized or non-randomized studies of healthcare interventions [19].

The AMSTAR-2 assessment was conducted independently by two reviewers (AMVB and EPV). Disagreements were resolved through discussion and consensus or, when necessary, through consultation with a third reviewer (CMA). For each included systematic review, the complete full-text publication and all available Supplementary Materials were examined before item-level judgments were assigned. Each of the 16 AMSTAR-2 items was rated as “Yes,” “Partial Yes,” “No,” or “Not Applicable,” according to the instrument guidance. The seven domains considered critical were protocol established before conduct of the review (item 2), comprehensiveness of the literature search (item 4), provision and justification of excluded studies (item 7), assessment of risk of bias in the included primary studies (item 9), appropriateness of meta-analytic methods (item 11), consideration of primary-study risk of bias when interpreting the findings (item 13), and assessment of publication bias when applicable (item 15).

AMSTAR-2 was not treated as a numerical scoring system. Overall confidence was determined from the pattern of critical and non-critical weaknesses rather than from an aggregate item score. High confidence indicated no or one non-critical weakness; moderate confidence indicated more than one non-critical weakness without a critical flaw; low confidence indicated one critical flaw with or without additional non-critical weaknesses; and critically low confidence indicated more than one critical flaw with or without additional non-critical weaknesses. “Partial Yes” judgments were retained as partial adherence to the corresponding item and were not converted into numerical points. The complete item-level assessment of all 16 AMSTAR-2 items, with critical domains explicitly identified and the resulting overall confidence rating for each systematic review, is presented in Supplementary Table S2.

### 2.7. Overlap Analysis of Primary Studies

To evaluate duplication of the underlying evidence across systematic reviews, a citation matrix was constructed by mapping the identifiable primary-study reports contributing to the eligible evidence in each review. Primary-study reports were identified from study-characteristics tables, included-study lists, and reference lists. Bibliographic records were cross-matched using author names, publication year, article title, journal, and DOI or PMID when available. Differences in spelling, diacritics, citation abbreviation, or publication year were reconciled when they clearly referred to the same publication, and exact duplicate citations were counted once.

The identifiable primary-study report was used as the unit of the citation matrix because the included systematic reviews did not consistently provide sufficient information to reconstruct underlying trial cohorts across multiple publications. Distinct publications were therefore retained as separate reports unless they could be unequivocally identified as the same bibliographic report; publications were not merged solely on the basis of shared authorship, intervention, or study setting. For systematic reviews with a broader scope, only reports contributing to the eligible primary-tooth pulpotomy evidence involving at least one calcium-silicate-based material were mapped.

The degree of overlap was quantified using the Corrected Covered Area (CCA) method [20]. The CCA was calculated as (N − r)/(rc − r), where N represents the total number of primary-study-report occurrences across the systematic reviews, r the number of unique identifiable primary-study reports, and c the number of systematic reviews. CCA values of 0–5% were interpreted as slight overlap, 6–10% as moderate, 11–15% as high, and >15% as very high overlap. The complete citation matrix is provided in Supplementary Table S4 to permit independent verification of the calculation.

### 2.8. Certainty of Evidence

Certainty of evidence was evaluated using the Grading of Recommendations Assessment, Development and Evaluation (GRADE) framework adapted for umbrella reviews [21,22]. Certainty was assessed separately for clinically relevant comparison–outcome pairs whenever clinical and radiographic outcomes could be distinguished in the included systematic reviews. Evidence derived predominantly from randomized clinical trials initially received a high-certainty rating; whereas, comparisons to which non-randomized intervention studies contributed materially started at moderate certainty.

Certainty could be downgraded by one level for a serious concern or by two levels for a very serious concern in the following domains: (1) methodological limitations, based on the AMSTAR-2 confidence of the contributing systematic reviews together with the reported risk of bias of their primary studies; (2) inconsistency, when reviews reported persistent differences in the direction or magnitude of effects for the same comparison, outcome, and comparable follow-up interval; (3) indirectness, when the population, intervention, comparator, or outcome did not correspond directly to the target clinical question; (4) imprecision, when estimates were based on few studies or events, had wide confidence intervals encompassing materially different clinical interpretations, or were available from only a limited evidence base; and (5) potential reporting bias, when selective reporting or publication bias was considered plausible on the basis of the information reported by the contributing reviews. Absence of a formal publication-bias test alone did not automatically result in downgrading.

Because multiple systematic reviews frequently included the same primary trials, primary-study overlap was additionally considered as an umbrella-review-specific indicator of evidence redundancy. Primary-study overlap did not automatically result in a separate downgrade for every outcome and was not double-counted when its consequences were already reflected in another domain. However, certainty was downgraded when apparent replication across reviews depended substantially on the same primary studies and therefore provided limited independent confirmation.

For evidence starting below high certainty because of material contribution from non-randomized studies, upgrading was considered when there was a large and consistent magnitude of effect, evidence of a dose–response relationship, or plausible residual confounding that would be expected to reduce rather than increase the observed effect. None of the evaluated comparison–outcome pairs fulfilled these criteria; therefore, no upgrading was applied. Final certainty was categorized as high, moderate, low, or very low.

### 2.9. Data Synthesis

A structured narrative synthesis was conducted to summarize and compare findings across the included systematic reviews. Particular attention was given to clinical and radiographic success, material comparisons, follow-up intervals, and pooled effect estimates reported by the original meta-analyses.

In addition, a functional meta-synthesis was performed as a structured review-level integration of the evidence. In the present umbrella review, this term refers to an analytical procedure designed to identify recurring clinical patterns across systematic reviews rather than to a statistical second-order meta-analysis. The systematic review constituted the unit of synthesis. For each review, findings were organized according to four predefined dimensions: (1) calcium-silicate-based material and comparator; (2) clinical or radiographic outcome; (3) follow-up interval; and (4) direction of the reported effect. Review-level findings were classified as favoring the calcium-silicate-based material, showing no statistically significant or clinically relevant difference between materials, favoring the comparator, or providing mixed or insufficient evidence.

The resulting review-level findings were compared across these dimensions to identify areas of convergence and discrepancy. A finding was considered a convergent evidence pattern when a similar direction of evidence was reported in at least two systematic reviews without a persistent opposing finding at comparable follow-up intervals. Findings reported by only one review were retained as comparison-specific observations and were not interpreted as cross-review convergence. Where results differed according to follow-up duration or outcome type, these differences were retained rather than collapsed into a single overall conclusion.

The AMSTAR-2 overall confidence ratings and the CCA were incorporated into the interpretation of the identified patterns to account for methodological limitations and redundancy of primary evidence across reviews. Effect estimates reported by the original meta-analyses were not statistically recombined. Because this umbrella review synthesized evidence at the systematic-review level, no de novo meta-analysis or meta-regression of primary studies was performed.

## 3. Results

### 3.1. Study Selection

**Figure 1:**
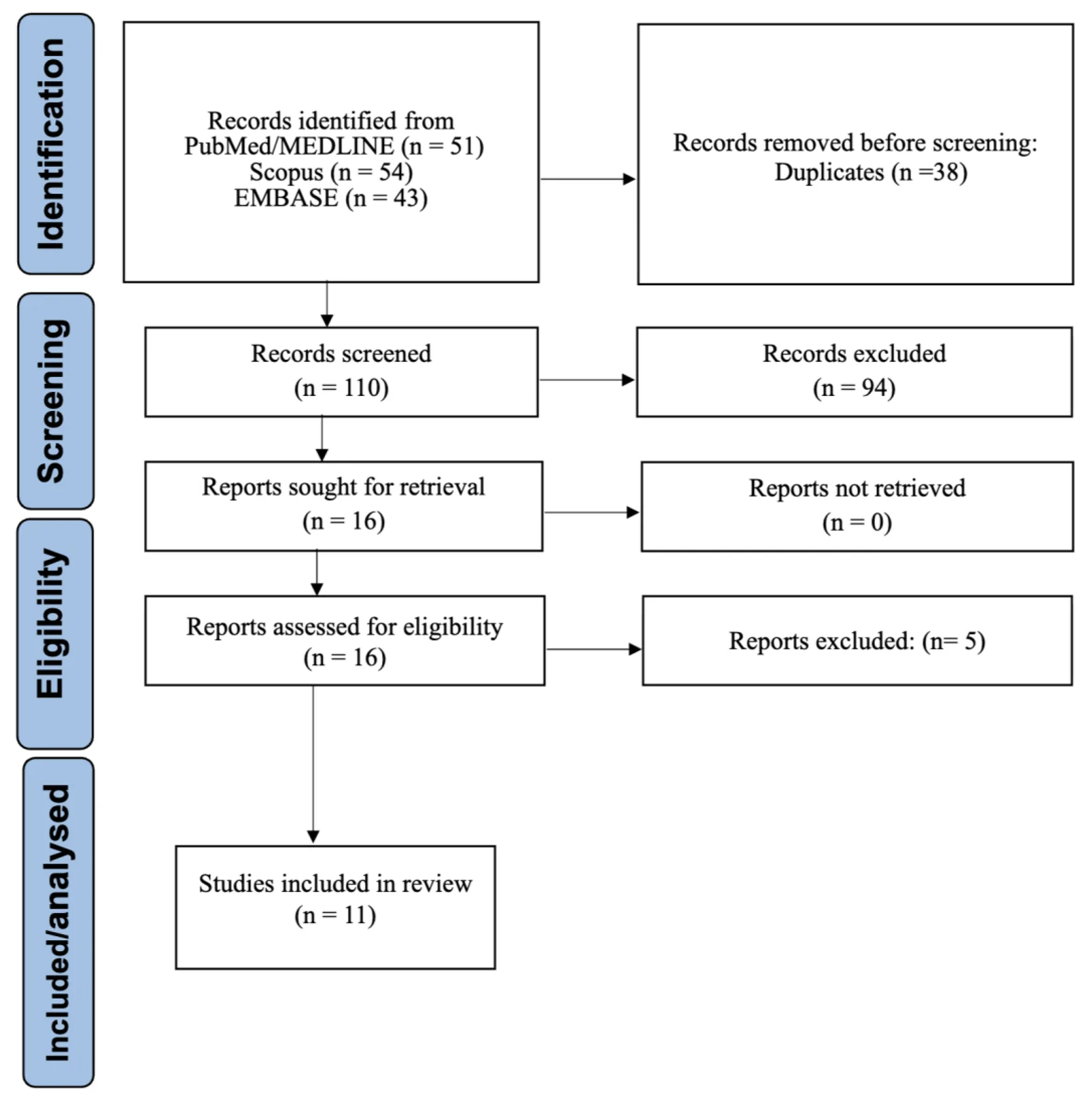
PRISMA flow diagram of the study selection process for the umbrella review.

The study selection process is illustrated in the PRISMA flow diagram (Figure 1). The database searches were conducted up to June 2026, with no restrictions on publication date. A total of 148 records were initially identified through the electronic database search. After removing 38 duplicate records, 110 unique records remained for title and abstract screening. Following application of the predefined eligibility criteria, 94 records were excluded, primarily because they were narrative or non-systematic reviews, focused on permanent teeth without separately extractable primary-tooth data, evaluated unrelated endodontic procedures, or did not assess pulpotomy outcomes involving calcium-silicate-based materials in primary teeth.

Sixteen articles were retrieved for full-text assessment. After detailed evaluation, five reviews were excluded. The exclusions reflected specific discrepancies between the review-level question or synthesis and the predefined eligibility criteria. These included reviews in which the relevant calcium-silicate-based evidence was embedded within a broader synthesis of pulp-treatment modalities rather than constituting a distinct material-centered primary-tooth pulpotomy synthesis; a review in which the material-specific comparison represented only a secondary component of a different clinical question; a review in which the separately reported primary-tooth pulpotomy evidence was synthesized primarily as material-specific success proportions rather than as an eligible comparative synthesis; and a review whose evidence base included non-comparative studies inconsistent with the predefined comparator requirement. Article-specific reasons for exclusion are reported in Supplementary Table S3. Eleven systematic reviews ultimately fulfilled all eligibility criteria and were included in the umbrella review [7,8,9,10,11,23,24,25,26,27,28].

### 3.2. Characteristics of the Included Systematic Reviews

The main characteristics of the included systematic reviews are summarized in Table 1. The 11 reviews were published between 2019 and 2025 and evaluated clinical and radiographic outcomes of pulpotomy procedures involving calcium-silicate-based materials in primary teeth [7,8,9,10,11,23,24,25,26,27,28]. Their scopes ranged from focused pairwise comparisons, particularly Biodentine versus MTA, to broader comparative syntheses evaluating calcium-silicate-based materials against conventional pulpotomy medicaments or other contemporary calcium-silicate formulations. One review had a broader vital pulp therapy scope involving primary and permanent dentitions; however, it organized the evidence around calcium-silicate-based cements and reported the primary-dentition evidence separately, allowing independent extraction of the eligible primary-tooth pulpotomy component [23].

The included reviews represented several recurring comparative evidence domains. Three systematic reviews were specifically dedicated to the comparison between Biodentine and MTA in primary-tooth pulpotomy [7,24,27]. Stringhini Junior et al. [7] included nine clinical trials, Nagendrababu et al. [24] included eight randomized controlled trials, and Lu et al. [27] included 11 randomized controlled trials in a more recent update extending follow-up assessment to 24 months. All three reported broadly comparable clinical and radiographic outcomes between the two materials, although Nagendrababu et al. [24] additionally concluded that the available evidence remained limited and of low certainty. Xavier et al. [23], within a broader calcium-silicate-based vital pulp therapy review, also provided a separately identifiable meta-analysis of ProRoot MTA versus Biodentine in primary teeth.

Comparisons involving conventional pulpotomy medicaments were addressed by several reviews. Shafaee et al. [8] compared Biodentine with MTA, formocresol, and ferric sulfate; whereas, Firoozi et al. [9] specifically updated the evidence comparing Biodentine with formocresol. Gopinath et al. [26] synthesized randomized trials comparing MTA, Biodentine, and calcium-enriched mixture cement with ferric sulfate. Bossù et al. [25] provided a broader material-centered systematic review of randomized comparative studies involving MTA, Biodentine, ferric sulfate, calcium hydroxide, and other pulpotomy agents; substantial methodological heterogeneity precluded quantitative pooling in that review.

Other reviews evaluated a broader range of contemporary calcium-silicate-based formulations. Silva et al. [10] analyzed randomized clinical trials comparing MTA with Biodentine, Portland cement, and TheraCal; whereas, Albernaz Neves et al. [11] compared materials classified by the authors as first- and second-generation bioceramics. Beldar et al. [28] evaluated MTA and other calcium-silicate-based materials against several conventional pulpotomy agents and reported clinical and radiographic outcomes across 30 included studies.

Xavier et al. [23] was the only included review with a broader vital pulp therapy scope encompassing both primary and permanent dentitions. Of the 40 trials included in that review, 22 involved primary teeth, all of which were primary molars. Two studies evaluated direct pulp capping and were not considered in the present umbrella review; the remaining 20 evaluated pulpotomy and provided separately identifiable clinical and radiographic outcomes and therefore constituted the eligible component incorporated into the present synthesis.

Across the 11 systematic reviews, the number of studies reported in the original reviews ranged from 8 to 41. For the overlap analysis, the number of eligible identifiable primary-study reports mapped per review ranged from 8 to 33. These counts differed for reviews with broader scopes or bibliographic duplication, as detailed in Table 1 and Supplementary Table S4. Individual primary-study reports were represented in more than one systematic review, as quantified in the overlap analysis reported in Section 3.4 and Supplementary Table S4.

The outcomes most frequently reported were clinical and radiographic success. Clinical success was generally characterized by the absence of spontaneous pain, swelling, sinus tract, tenderness, or pathological mobility; whereas, radiographic success was generally based on the absence of furcal or periapical radiolucency, pathological internal or external root resorption, and other radiographic signs of treatment failure. Definitions and follow-up intervals varied across reviews and were retained according to the criteria reported by the respective review authors.

### 3.3. Methodological Quality of the Included Reviews (AMSTAR-2)

The methodological quality of the 11 included systematic reviews was assessed using AMSTAR-2. Table 2 provides a condensed summary of selected methodological domains and overall confidence ratings; whereas, Supplementary Table S2 presents the complete judgments for all 16 AMSTAR-2 items for each review, with the seven critical domains explicitly identified. Overall confidence ratings were derived from the pattern of critical and non-critical weaknesses according to AMSTAR-2 guidance and were not based on a numerical score.

Overall confidence was rated as critically low for Stringhini Junior et al. [7], Bossù et al. [25], Lu et al. [27], and Beldar et al. [28]; low for Shafaee et al. [8], Firoozi et al. [9], Albernaz Neves et al. [11], Xavier et al. [23], and Gopinath et al. [26]; and moderate for Silva et al. [10] and Nagendrababu et al. [24].

Two reviews provided moderate overall confidence: Silva et al. [10] and Nagendrababu et al. [24]. Both reported protocol registration, duplicate review procedures, formal risk-of-bias assessment, appropriate quantitative synthesis, and structured evaluation of evidence certainty. Remaining limitations concerned aspects such as search completeness, reporting of primary-study funding, and other review-level reporting requirements.

Five reviews were rated as providing low confidence [8,9,11,23,26]. Their principal critical weaknesses differed across reviews and included absence of a prespecified protocol, incomplete reporting of excluded studies, lack of publication-bias assessment, incomplete duplicate review procedures, or limitations in risk-of-bias appraisal. These deficiencies reduced confidence in the review-level estimates despite generally appropriate quantitative synthesis.

Four reviews were rated as critically low confidence [7,25,27,28] because multiple critical weaknesses were identified. These included combinations of absent protocol registration, limited literature searching, incomplete reporting of excluded studies, insufficiently specified or methodologically problematic quantitative synthesis, and inadequate assessment of publication bias. Overall, two reviews were rated moderate, five low, and four critically low; none achieved high confidence. Detailed item-level judgments are provided in Supplementary Table S2.

### 3.4. Overlap of Primary-Study Reports (Corrected Covered Area—CCA)

The complete citation matrix of primary-study reports across the 11 included systematic reviews is provided in Supplementary Table S4. Across the 11 reviews, 78 unique identifiable primary-study reports (r) accounted for 180 primary-study-report occurrences (N). With 11 systematic reviews (c), the resulting CCA was 13.1% [CCA = (180 − 78)/((78 × 11) − 78)], corresponding to high overlap according to the predefined thresholds.

The number of eligible primary-study reports represented per review ranged from 8 to 33. Repeated representation of the same reports was particularly frequent among reviews evaluating MTA and Biodentine. The citation matrix was constructed at the report level; therefore, the 78 unique entries represent identifiable primary-study reports rather than reconstructed unique trial cohorts.

### 3.5. Quantitative Findings from Meta-Analyses

Ten of the 11 included systematic reviews performed quantitative synthesis; whereas, Bossù et al. [25] used a descriptive synthesis because of substantial clinical and methodological heterogeneity. The quantitative findings reported below represent review-level estimates reproduced from the original meta-analyses; no second-order statistical pooling was performed in the present umbrella review. Detailed effect estimates and measures of precision are presented in Table 3.

The most extensively investigated comparison was MTA versus Biodentine, which was quantitatively evaluated in eight systematic reviews [7,8,10,11,23,24,27,28]. Nagendrababu et al. [24] reported no significant differences in clinical success at 6 months (RR = 1.00; 95% CI: 0.97–1.02), 12 months (RR = 1.00; 95% CI: 0.96–1.05), or 18 months (RR = 1.00; 95% CI: 0.93–1.08); trial sequential analysis did not establish firm evidence, and the certainty of evidence was rated as low. Lu et al. [27] reported no significant difference at 24 months for either clinical success (RR = 1.00; 95% CI: 0.94–1.07) or radiographic success (RR = 1.01; 95% CI: 0.94–1.08). Stringhini Junior et al. [7], Xavier et al. [23], and Albernaz Neves et al. [11] also reported estimates close to 1.00 across the evaluated follow-up intervals. Shafaee et al. [8], who analyzed failure rather than success, reported significantly higher radiographic failure with Biodentine than with MTA at 6 and 9–12 months; whereas, clinical failure did not differ substantially.

For Biodentine versus formocresol, Firoozi et al. [9] reported higher radiographic success with Biodentine at 6 months (RR = 1.12; 95% CI: 1.02–1.23) and 12 months (RR = 1.14; 95% CI: 1.037–1.25). The primary clinical analysis yielded RR = 1.05 (95% CI: 1.003–1.097) at 12 months; after influence analysis, the estimate was RR = 1.04 (95% CI: 0.98–1.11), and trial sequential analysis did not establish firm evidence for a clinical difference. Shafaee et al. [8] reported no substantial clinical or radiographic failure difference between Biodentine and formocresol.

For comparisons involving ferric sulfate, Gopinath et al. [26] reported no significant difference between MTA and ferric sulfate at 24 months for clinical success (RR = 0.98; 95% CI: 0.15–6.34) or radiographic success (RR = 0.74; 95% CI: 0.23–2.43). Biodentine and ferric sulfate also showed no significant difference at 6 months for clinical success (RR = 1.00; 95% CI: 0.95–1.05) or radiographic success (RR = 0.99; 95% CI: 0.88–1.11). For CEM versus ferric sulfate, the radiographic estimate at 6 months was RR = 0.25 (95% CI: 0.03–2.22).

Other quantitative comparisons were less frequently represented. Silva et al. [10] reported no significant clinical differences between MTA and Biodentine and higher radiographic success with MTA at 12 months, with no significant radiographic difference at 24 months. The same review reported no significant differences for MTA versus Portland cement at 24 months or MTA versus TheraCal at 12 months. Albernaz Neves et al. [11] reported no significant clinical or radiographic differences between first- and second-generation bioceramics throughout the 24 months. Beldar et al. [28] reported pooled MTA clinical and radiographic success rates of 97.02% and 94.21%, respectively, together with additional material-specific comparative estimates.

**Figure 2:**
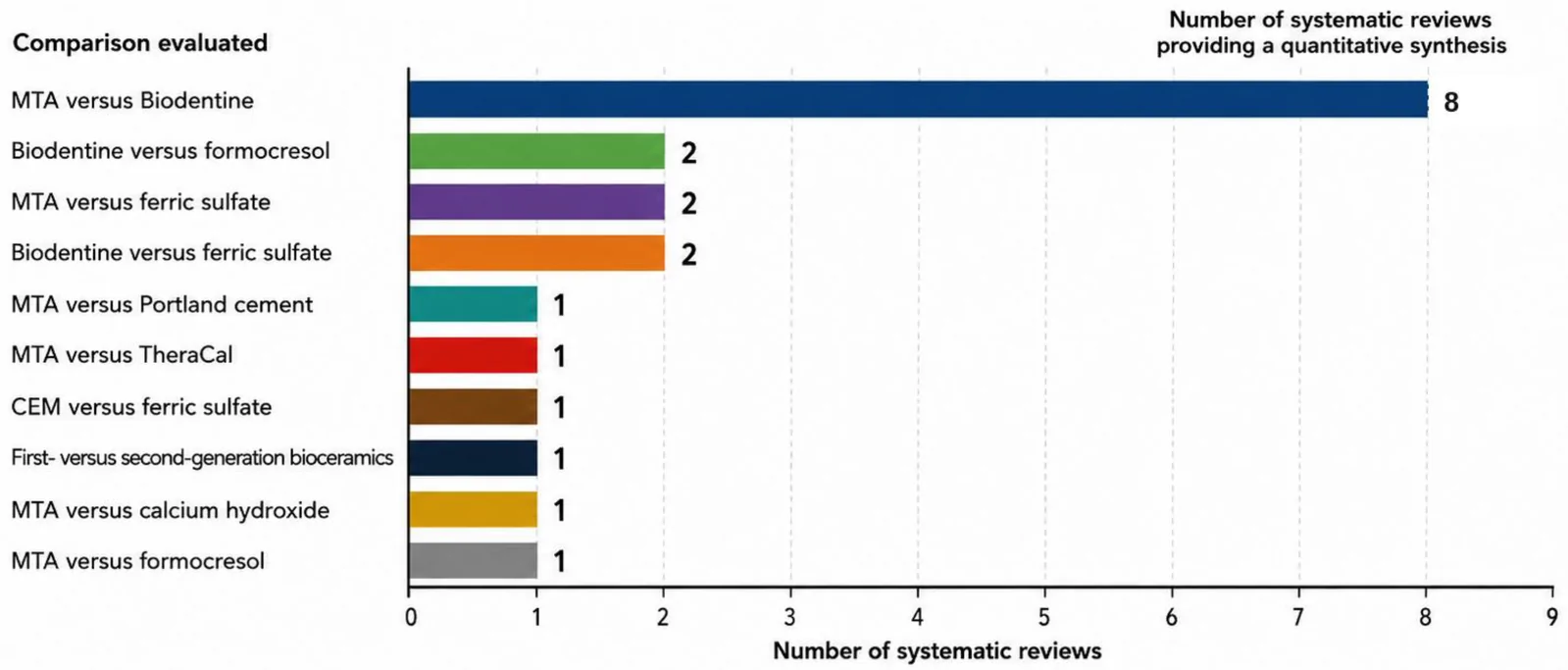
Summary evidence map of quantitative comparisons reported in systematic reviews and meta-analyses evaluating pulpotomy materials in vital primary teeth. The bars indicate the number of systematic reviews that provided a quantitative synthesis for each material comparison. MTA versus Biodentine was the most frequently evaluated comparison, with quantitative evidence reported in eight systematic reviews [7,8,10,11,23,24,27,28]. Other recurrent comparisons included Biodentine versus formocresol [8,9], MTA versus ferric sulfate [26,28], and Biodentine versus ferric sulfate [8,26]. Additional comparisons were represented by a single systematic review. Across the included reviews, primary-study-report overlap was high (CCA = 13.1%). MTA, mineral trioxide aggregate; CEM, calcium-enriched mixture cement.

Table 3 presents the principal quantitative estimates reported by the included meta-analyses, and Figure 2 shows the distribution of quantitative evidence across material comparisons.

### 3.6. Functional Meta-Synthesis of Evidence

The functional meta-synthesis identified the strongest cross-review directional consistency for the MTA–Biodentine comparison. Most contributing reviews reported no significant clinical or radiographic differences, although Shafaee et al. [8] and Silva et al. [10] reported radiographic findings favoring MTA at selected follow-up intervals.

Review-level findings for Biodentine versus formocresol were not fully concordant. Shafaee et al. [8] reported no substantial clinical or radiographic failure difference; whereas, Firoozi et al. [9] reported higher radiographic success with Biodentine at 6 and 12 months. Comparisons involving ferric sulfate were reported in fewer reviews and varied according to material and outcome. Evidence for Portland cement, TheraCal, CEM, and materials classified as second-generation bioceramics was represented by fewer comparison-specific review-level syntheses.

**Figure 3:**
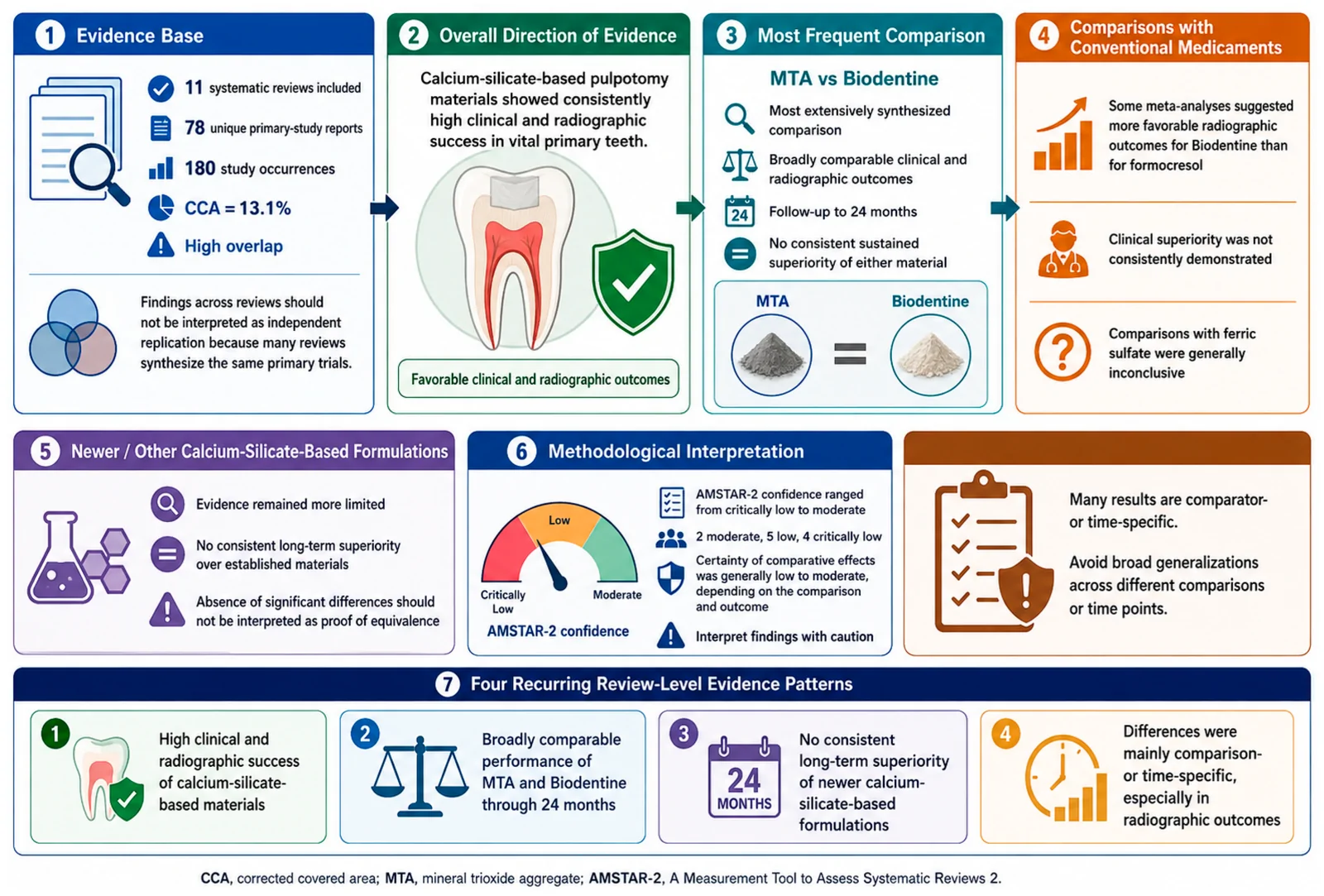
Graphical integration of the review-level evidence synthesized in the umbrella review. The figure summarizes the direction and consistency of the principal comparison-specific findings together with methodological confidence, certainty of evidence, and primary-study overlap. Eleven systematic reviews contributed 78 unique primary-study reports and 180 primary-study-report occurrences, yielding a CCA of 13.1% (high overlap).

Figure 3 displays these comparison-specific patterns together with AMSTAR-2 confidence, GRADE-informed certainty, and the observed primary-study-report overlap.

### 3.7. Additional Outcomes

Additional outcomes defined in the methodology included treatment failure, adverse events, and the need for retreatment. These outcomes were reported less consistently than clinical and radiographic success across the 11 included systematic reviews. In most reviews, failure was incorporated into the definitions of clinical or radiographic success rather than analyzed as a separate outcome; whereas, dedicated reporting of adverse events and retreatment was uncommon [7,8,9,10,11,23,24,25,26,27,28].

Clinical failure was generally defined by signs or symptoms such as spontaneous pain, swelling, fistula or sinus tract, tenderness, or pathological mobility. Radiographic failure commonly included furcal or periapical radiolucency, pathological external root resorption, periodontal-ligament changes, and, in some studies, internal root resorption [7,10,11,23,24,25,26,27,28]. Radiographic definitions were not uniform across reviews. Pulp canal obliteration and some forms of non-perforating or self-limiting internal root resorption were not consistently classified as treatment failure. Xavier et al. [23] reported variation in these definitions across the contributing primary trials.

Lu et al. [27] separately analyzed root resorption and pulp canal obliteration in the comparison between Biodentine and MTA. At follow-up intervals of 6, 12, and ≥18 months, no significant differences between the two materials were reported for either outcome.

Gopinath et al. [26] discussed adverse effects associated with bioactive endodontic materials and ferric sulfate, including occasional canal obliteration and delayed pulpal healing associated with ferric sulfate, but did not provide a separate pooled comparative analysis of adverse events. Bossù et al. [25] discussed reported cytotoxic and carcinogenic concerns associated with formocresol but did not provide comparative incidence estimates for adverse events. No included review provided a pooled comparison of adverse-event incidence across all eligible calcium-silicate-based materials.

Retreatment was rarely reported as a distinct review-level outcome. Tooth extraction or additional endodontic intervention was generally incorporated into treatment-failure reporting. No separate pooled review-level comparison of retreatment requirements among the eligible materials was identified.

### 3.8. Certainty of Evidence

The GRADE-informed certainty assessment was conducted separately for the principal comparison–outcome pairs for which review-level evidence could be meaningfully distinguished (Table 4). Clinical and radiographic outcomes were evaluated independently when the direction or consistency of effects differed between these outcome domains.

For MTA versus Biodentine, certainty was rated as low for both clinical and radiographic success. For clinical success, downgrading reflected methodological limitations among the contributing reviews and redundancy of primary evidence across reviews. Radiographic evidence was also rated as low, with inconsistency at selected follow-up intervals contributing to the judgment. Nagendrababu et al. [24] independently rated the underlying evidence as low quality, and trial sequential monitoring boundaries for firm evidence were not reached.

For Biodentine versus formocresol, certainty was rated as low for both clinical and radiographic outcomes. The clinical judgment reflected methodological limitations and limited comparison-specific evidence. For radiographic success, inconsistency across reviews was additionally considered because Shafaee et al. [8] reported no substantial difference; whereas, Firoozi et al. [9] reported higher radiographic success with Biodentine at 6 and 12 months.

For Biodentine versus ferric sulfate, certainty was rated as low for the available clinical and radiographic evidence. MTA versus ferric sulfate was rated as very low because of methodological limitations, inconsistency, and serious imprecision. CEM versus ferric sulfate was also rated as very low because of methodological limitations and very serious imprecision associated with sparse comparison-specific evidence [26].

MTA versus Portland cement and MTA versus TheraCal were each rated as low certainty [10]. Evidence comparing first- and second-generation bioceramics was also rated as low certainty [11]. The 14 studies ultimately included in Albernaz Neves et al. [11] were reported as randomized controlled clinical trials, although that review’s eligibility criteria permitted both randomized and non-randomized intervention studies.

Across the 10 comparison–outcome categories summarized in Table 4, eight were rated as low certainty and two as very low certainty. No comparison–outcome category was rated as moderate or high certainty, and no category fulfilled the predefined criteria for upgrading.

## 4. Discussion

The present umbrella review synthesized 11 systematic reviews evaluating calcium-silicate-based materials for pulpotomy in vital primary teeth [7,8,9,10,11,23,24,25,26,27,28]. Its principal contribution is to clarify the consistency, methodological confidence, certainty, and independence of the available review-level evidence rather than to generate new pooled efficacy estimates. This distinction is particularly relevant because primary-study overlap was high (CCA = 13.1%) and AMSTAR-2 confidence ranged from critically low to moderate.

MTA and Biodentine remain the most extensively investigated materials. Eight systematic reviews provided quantitative evidence for this comparison, [7,8,10,11,23,24,27,28] while Bossù et al. [25] provided additional descriptive evidence within a broader material comparison. Most syntheses reported comparable clinical and radiographic outcomes over follow-up periods extending to 24 months. Although Shafaee et al. [8] and Silva et al. [10] identified radiographic differences favoring MTA at selected follow-up intervals, these findings were not consistently reproduced or sustained at longer follow-up. Nagendrababu et al. [24] likewise found no significant clinical or radiographic differences through 18 months and rated the underlying evidence as low certainty, while trial sequential analysis indicated that firm evidence had not been reached. More recently, Lu et al. [27] extended the comparison through 24 months without demonstrating consistent superiority of either material. Taken together, the review-level evidence supports the conclusion that MTA and Biodentine perform broadly similarly, but the extensive reuse of the same primary trials means that agreement among multiple reviews should not be interpreted as independent confirmation. This interpretation is consistent with contemporary evidence-based guidance supporting calcium-silicate cements for primary-tooth pulpotomy [29].

Evidence concerning newer or less extensively studied calcium-silicate-based formulations remains less mature. Reviews evaluating Portland cement, TheraCal-based products, calcium-enriched mixture cement, and materials classified as second-generation bioceramics generally did not demonstrate consistent superiority over established calcium-silicate materials [10,11,26]. Recent randomized trials provide additional clinical context. Talekar et al. [30] reported favorable outcomes over 24 months across three bioceramic calcium-silicate cements, Alnassar et al. [31] observed favorable 12-month outcomes with both MTA and bioceramic putty in symptomatic primary molars, and Alqahtani et al. [32] reported comparable clinical and radiographic outcomes for NeoPUTTY and NeoMTA 2. Hassanpour et al. [33] similarly found no significant difference between TheraCal and MTA at 12 months. These results are encouraging, but the smaller evidence base, limited number of direct comparisons, and imprecision associated with several newer formulations preclude conclusions of either superiority or therapeutic equivalence.

Comparisons with conventional pulpotomy medicaments showed greater variability. For Biodentine versus formocresol, Shafaee et al. [8] found no substantial difference in clinical or radiographic failure; whereas, Firoozi et al. [9] reported a radiographic advantage for Biodentine at 6 and 12 months without robust evidence of clinical superiority. Thus, a possible radiographic advantage of Biodentine over formocresol was not consistently demonstrated across reviews. Evidence involving ferric sulfate was less consistent. Gopinath et al. [26] found no significant differences in the available comparisons of MTA or Biodentine with ferric sulfate; whereas, Beldar et al. [28] reported more favorable results for calcium-silicate-based materials within a review judged to have critically low methodological confidence. Consequently, certainty remained low for Biodentine versus ferric sulfate and very low for MTA versus ferric sulfate. Bossù et al. [25] also provided descriptive evidence across several conventional pulpotomy agents but did not perform quantitative pooling because of substantial heterogeneity. These findings indicate that any apparent advantage of calcium-silicate-based materials over conventional medicaments is likely to be comparator- and outcome-specific rather than uniform across all clinical and radiographic endpoints. Petel et al. [34], for example, reported comparable long-term outcomes between Portland cement and formocresol over two to four years. Clinical guidelines provide additional context for medicament selection [29], but such recommendations incorporate biological, safety, practical, and patient-related considerations beyond the comparative evidence synthesized here.

Follow-up duration partly contributed to variation among review-level findings. Some statistically significant differences appeared at intermediate assessments, particularly around 12 months, but were generally not sustained at longer follow-up. Such variation may reflect sample size, outcome definitions, radiographic interpretation, attrition, or timing of pulpal and periradicular changes rather than persistent differences in material effectiveness. Individual trials also illustrate this uncertainty. Tungjitphianpong et al. [35] reported no significant differences among MTA, Biodentine, and acemannan at 24 months in a randomized trial of partial pulpotomy; whereas, Wassel et al. [36] reported acceptable one-year outcomes with TheraCal PT within a broader vital pulp therapy comparison. Gisour et al. [37] similarly found no significant 12-month differences among formocresol, Portland cement, and NeoMTA Plus despite numerical variation in radiographic outcomes. These studies provide useful clinical context, but differences in procedures, populations, and follow-up should be considered when extrapolating their findings to full coronal pulpotomy.

An important finding of the present umbrella review was the extent of redundancy in the underlying evidence. Across the 11 included reviews, 78 unique primary-study reports accounted for 180 study occurrences, yielding a CCA of 13.1%, classified as high overlap. Redundancy was particularly evident in the MTA–Biodentine evidence base, where the same trials were repeatedly incorporated into focused and broader systematic reviews. Consequently, apparently consistent results across several meta-analyses do not represent an equivalent number of independent demonstrations of treatment effect. This issue is especially relevant when newer systematic reviews update an earlier clinical question without replacing the underlying trials. The functional meta-synthesis therefore treated concordance across reviews as convergence of review-level findings rather than independent replication.

Methodological quality further limits the strength of comparative conclusions. Of the 11 reviews, two were rated as providing moderate AMSTAR-2 confidence, five as low confidence, and four as critically low confidence; none achieved high confidence. Limitations included insufficiently comprehensive searches in some reviews, absence of prospective protocols, incomplete reporting of excluded studies, inadequate consideration of primary-study risk of bias, inconsistent assessment of publication bias, and limited certainty-of-evidence evaluation. Accordingly, the GRADE-informed assessment classified the principal MTA–Biodentine and Biodentine–formocresol comparisons as low certainty, with very low certainty for several less frequently studied comparisons. The consistency of treatment success across reviews is therefore reassuring, but confidence in small differences between individual materials remains substantially weaker than confidence in the general effectiveness of calcium-silicate-based pulpotomy materials.

From a clinical perspective, the available evidence does not support preferential selection of a pulpotomy material solely on the basis of small or isolated differences reported in individual meta-analyses. Material selection should instead consider the certainty and independence of the comparative evidence together with tooth- and patient-related factors, handling characteristics, availability, cost, clinician experience, and applicable clinical guidance. Additional comparative evidence is particularly needed before preferential recommendations can be made for less extensively studied calcium-silicate-based formulations.

This umbrella review has several strengths. It integrates the available systematic-review evidence while explicitly evaluating methodological quality, quantifying primary-study overlap, distinguishing clinical from radiographic outcomes, and assessing certainty at the comparison–outcome level. The citation-matrix approach additionally prevents repeated synthesis of the same trials from being interpreted as independent confirmation. Nevertheless, important limitations remain. Despite the inclusion of 11 systematic reviews, the evidence was concentrated in a relatively limited and highly reused set of primary trials, methodological confidence was predominantly low or critically low, and several material comparisons remained supported by sparse or imprecise evidence. Definitions of clinical and radiographic success and follow-up intervals were not completely uniform, and adverse events, retreatment, and patient-centered outcomes were inconsistently reported. These limitations also precluded robust comparative conclusions regarding safety. Future randomized trials should prioritize adequately powered direct comparisons, standardized outcome definitions, follow-up of at least 24 months, transparent reporting of attrition and adverse events, and clinically relevant patient-centered outcomes. Future systematic reviews should also avoid unnecessary duplication of established questions and clearly establish whether additional primary evidence materially changes previous conclusions.

## 5. Conclusions

Calcium-silicate-based materials are associated with high clinical and radiographic success in vital primary-tooth pulpotomy, but the available review-level evidence does not establish sustained superiority of one contemporary formulation over another. Comparative findings involving conventional medicaments and less extensively studied calcium-silicate-based materials remain outcome- and comparator-specific and are supported by limited-certainty evidence. Because methodological confidence ranged from critically low to moderate and primary-study overlap was high, the evidence supports the effectiveness of calcium-silicate-based materials as a class more strongly than preferential selection of any individual material.

## Figures and Tables

**Table 1:** Characteristics of the systematic reviews included in the umbrella review.

Review	Review Question/Scope	Databases	Study Design Included	No. Studies Reported in Review/Eligible Primary-Study Reports Mapped in CCA	Intervention/Comparison	Main Synthesis Approach	Comparator-Specific Conclusion(s)
Stringhini Junior et al. 2019 [7].	Compared clinical and radiographic success of Biodentine versus MTA for pulpotomy in primary teeth.	Nine databases, including PubMed, Lilacs, Cochrane, Embase, Scopus, Web of Science, CINAHL, EBSCO, and ClinicalTrials.gov	Clinical trials.	9/9	Biodentine vs. MTA.	Meta-analysis at 6, 12, and 18 months.	Biodentine vs. MTA: No statistically significant differences in clinical or radiographic success were demonstrated at 6, 12, or 18 months.
Shafaee et al. 2019 [8].	Compared Biodentine with MTA, formocresol, and ferric sulfate regarding clinical and radiographic failure after pulpotomy in primary teeth.	MEDLINE, Embase, Cochrane Central Register of Controlled Trials, and Web of Science; plus trial registry and references.	Randomized controlled trials.	10/10	Biodentine vs. MTA, formocresol, and ferric sulfate.	Meta-analysis by follow-up period.	Biodentine vs. MTA: Clinical failure did not differ substantially; radiographic failure was higher with Biodentine at 6 and 9–12 months. Biodentine vs. formocresol: No substantial clinical or radiographic failure differences. Biodentine vs. ferric sulfate: No substantial clinical or radiographic failure differences.
Firoozi et al. 2022 [9].	Updated meta-analysis comparing Biodentine and formocresol in carious primary molar pulpotomy.	MEDLINE, CENTRAL, Web of Science, Scopus, Google Scholar, manual searches.	Randomized controlled trials.	9/9	Biodentine vs. formocresol.	Meta-analysis plus trial sequential analysis.	Biodentine vs. formocresol: Radiographic success favored Biodentine at 6 and 12 months; robust clinical superiority was not demonstrated after sensitivity and trial sequential analyses.
Silva et al. 2024 [10].	Assessed success of primary tooth pulpotomy using MTA compared with other calcium-silicate cements.	PubMed, Cochrane Library, Scopus, Web of Science, Embase, ScienceDirect, and OpenGrey.	Randomized clinical trials.	26/26	MTA vs. Biodentine, Portland cement, and TheraCal.	Meta-analysis by material and follow-up.	MTA vs. Biodentine: No significant clinical differences; radiographic success favored MTA at 12 months but not at 24 months. MTA vs. Portland cement: No significant difference at 24 months. MTA vs. TheraCal: No significant difference at 12 months.
Albernaz Neves et al. 2025 [11].	Compared first- and second-generation bioceramic materials in primary dentition pulpotomies.	PubMed, MEDLINE, CENTRAL, Web of Science, SciELO, LILACS, and TRIP	Randomized controlled clinical trials	14/14	Second-generation bioceramics vs. MTA (first-generation bioceramic).	Random-effects meta-analysis with subgroup analyses.	First- vs. second-generation bioceramics: No significant differences in clinical or radiographic success were demonstrated through 24 months.
Xavier et al. 2024 [23].	Evaluated clinical, radiographic, and histological outcomes of calcium-silicate-based cements in vital pulp therapy in primary and permanent dentitions; only primary-tooth pulpotomy evidence was eligible for the present umbrella review	PubMed, LILACS, and Cochrane Collaboration; manual journal and reference searches; author contact	Randomized controlled trials	40 overall/20 eligible	Comparisons among different calcium-silicate-based cements, principally ProRoot MTA versus Biodentine	Separate synthesis by dentition and procedure; meta-analysis of ProRoot MTA versus Biodentine in primary teeth	ProRoot MTA vs. Biodentine in primary-tooth pulpotomy: No significant differences in clinical or radiographic success were demonstrated at 6 months or 2 years.
Nagendrababu et al. 2019 [24].	Compared clinical and radiographic success of Biodentine and MTA in vital primary molar pulpotomy.	PubMed, EBSCOhost, and Scopus; supplemented by clinical trial registries, reference lists, and textbooks.	Randomized controlled trials.	8/8	Biodentine vs. MTA.	Random-effects meta-analysis, trial sequential analysis, and GRADE.	Biodentine vs. MTA: No significant differences in clinical or radiographic success were demonstrated at 6, 12, or 18 months; the evidence was rated as low quality, and trial sequential analysis did not establish firm evidence.
Bossù et al. 2020 [25].	Compared different pulp-dressing materials for pulpotomy of primary teeth to identify a preferred material.	MEDLINE/PubMed; supplemented by reference searching.	Randomized comparative clinical studies.	41 overall/33 eligible	MTA, Biodentine, ferric sulfate, calcium hydroxide, and other pulpotomy agents.	Descriptive qualitative synthesis; meta-analysis was not performed because of heterogeneity.	MTA vs. other pulpotomy agents: MTA showed the most favorable overall descriptive profile. Biodentine and ferric sulfate: Favorable outcomes were reported. Calcium hydroxide: Poorer outcomes were reported. No pooled pairwise estimates were available because meta-analysis was not performed.
Gopinath et al. 2022 [26].	Compared bioactive endodontic materials with ferric sulfate for pulpotomy in primary molars.	PubMed, EBSCOhost, ProQuest, and Scopus; supplemented by reference lists, textbooks, and gray literature.	Randomized controlled trials.	11/11	MTA, Biodentine, and CEM vs. ferric sulfate.	Random-effects meta-analysis by material and follow-up period.	MTA vs. ferric sulfate: No significant clinical or radiographic difference at 24 months. Biodentine vs. ferric sulfate: No significant clinical or radiographic difference at 6 months. CEM vs. ferric sulfate: No significant radiographic difference at 6 months; comparison-specific evidence was sparse.
Lu et al. 2025 [27].	Compared the efficacy of Biodentine and MTA in primary-tooth pulpotomy, including clinical, radiographic, and selected endodontic outcomes.	PubMed, Scopus, Cochrane Library, and Web of Science.	Randomized controlled trials.	11/11	Biodentine vs. MTA.	Random-effects meta-analysis by follow-up period, with additional analyses of root resorption and pulp canal obliteration.	Biodentine vs. MTA: No significant differences in clinical or radiographic success were demonstrated through 24 months.
Beldar et al. 2025 [28].	Evaluated MTA and other calcium-silicate-based materials compared with conventional pulpotomy materials in primary teeth.	PubMed, Cochrane Library, Google Scholar, and Semantic Scholar.	Randomized controlled trials	30 reported/29 identifiable	MTA and other calcium-silicate-based materials, including CEM and Biodentine, vs. formocresol, ferric sulfate, calcium hydroxide, and other materials.	Meta-analysis of clinical and radiographic success with heterogeneity and publication-bias assessments.	MTA vs. formocresol, ferric sulfate, and calcium hydroxide: The review authors reported higher clinical and radiographic success with MTA. MTA vs. CEM and other calcium-silicate cements: Comparable success was reported. MTA vs. Biodentine: Comparative reporting was not fully consistent within the review.

MTA, mineral trioxide aggregate; CEM, calcium-enriched mixture cement; CENTRAL, Cochrane Central Register of Controlled Trials; LILACS, Literatura Latino-Americana e do Caribe em Ciências da Saúde; TRIP, Turning Research Into Practice; SciELO, Scientific Electronic Library Online; CINAHL, Cumulative Index to Nursing and Allied Health Literature; GRADE, Grading of Recommendations Assessment, Development and Evaluation. Terminology describing first- and second-generation bioceramics was retained when reflecting the classification used in the original systematic review.

**Table 2:** AMSTAR-2 appraisal of the 11 included systematic reviews.

Review	Protocol Registered	Comprehensive Search	Study Selection/Extraction in Duplicate	Risk of Bias of Primary Studies	Meta-Analysis Appropriate	Certainty/GRADE	Overall AMSTAR-2 Rating
Stringhini Junior et al. [7]	No	Partial	Yes	Yes (Cochrane)	Insufficiently reported	Not reported	Critically low
Shafaee et al. [8]	No	Partial	Yes	Yes	Yes	GRADE	Low
Firoozi et al. [9]	PROSPERO	Partial	Yes	Yes	Yes	No formal GRADE	Low
Silva et al. [10]	PROSPERO	Partial	Yes	Yes	Yes	GRADE	Moderate
Albernaz Neves et al. [11]	PROSPERO	Partial	Yes	Yes	Yes	No formal GRADE	Low
Xavier et al. [23]	PROSPERO	Partial	Not clearly reported	Partial	Yes	Not reported	Low
Nagendrababu et al. [24]	PROSPERO	Partial	Yes	Yes	Yes	GRADE	Moderate
Bossù et al. [25]	No	Limited	Selection yes; extraction unclear	Yes	NA	Not reported	Critically low
Gopinath et al. [26]	PROSPERO	Partial	Yes	Yes	Yes	No formal GRADE	Low
Lu et al. [27]	No	Partial	Yes	Yes	Yes	Not reported	Critically low
Beldar et al. [28]	PROSPERO	Partial	Not clearly reported	Yes	No/major concerns	Not reported	Critically low

Item-level judgments were assigned after examination of the complete full-text publication and all available Supplementary Materials for each systematic review. An item was rated “No” when the required methodological information could not be identified in these sources or when the reported methods did not satisfy the corresponding AMSTAR-2 criterion; “Partial Yes” was used when the criterion was only partially fulfilled.

**Table 3:** Summary of quantitative findings across included meta-analyses.

Review	Comparison Evaluated	Follow-Up Intervals	Principal Quantitative Findings as Reported by the Review
Stringhini Junior et al. [7]	Biodentine vs. MTA	6, 12, 18 months	Clinical RR: 0.99 (95% CI: 0.96–1.02), 1.01 (0.97–1.04), and 0.98 (0.92–1.05), respectively. Radiographic RR: 0.96 (0.92–1.00), 0.97 (0.92–1.02), and 1.00 (0.91–1.10), respectively. No significant differences.
Shafaee et al. [8]	Biodentine vs. MTA; Biodentine vs. formocresol; Biodentine vs. ferric sulfate	<3 to ≥18 months	Meta-analysis was based on clinical and radiographic failure. Radiographic failure was significantly higher with Biodentine than with MTA at 6 and 9–12 months; clinical failure did not differ substantially. No substantial clinical or radiographic failure differences were found for Biodentine vs. formocresol or Biodentine vs. ferric sulfate.
Firoozi et al. [9]	Biodentine vs. formocresol	6 and 12 months	Clinical: 6-month RR = 1.03 (95% CI: 0.995–1.062); 12-month RR = 1.05 (1.003–1.097), reduced to RR = 1.04 (0.98–1.11) after influence analysis. Radiographic: 6-month RR = 1.12 (1.02–1.23); 12-month RR = 1.14 (1.037–1.25), favoring Biodentine. TSA supported the radiographic but not the clinical conclusion.
Silva et al. [10]	MTA vs. Biodentine; MTA vs. Portland cement; MTA vs. TheraCal	6–24 months	No significant clinical difference between MTA and Biodentine at any evaluated interval. Radiographic success favored MTA at 12 months only, with no significant difference at 24 months. No significant differences were identified for MTA vs. Portland cement at 24 months or MTA vs. TheraCal at 12 months.
Albernaz Neves et al. [11]	First- vs. second-generation bioceramics, including MTA vs. Biodentine	3–24 months	For MTA vs. Biodentine, clinical RRs ranged from 0.98 to 1.01 and radiographic RRs from 0.96 to 1.01 across follow-up intervals; all comparisons were non-significant.
Xavier et al. [23]	ProRoot MTA vs. Biodentine in primary teeth	6 months and 2 years	At 6 months: clinical RR = 0.994 (95% CI: 0.952–1.037); radiographic RR = 1.002 (0.966–1.040). At 2 years: clinical RR = 1.011 (0.966–1.058); radiographic RR = 1.007 (0.931–1.089). No significant differences.
Nagendrababu et al. [24]	Biodentine vs. MTA	6, 12, 18 months	Clinical RR: 1.00 (95% CI: 0.97–1.02), 1.00 (0.96–1.05), and 1.00 (0.93–1.08), respectively. Radiographic RR: 0.99 (0.96–1.02), 1.02 (0.47–2.21), and 1.02 (0.91–1.15), respectively. TSA did not establish firm evidence; certainty was rated low.
Gopinath et al. [26]	MTA vs. ferric sulfate; Biodentine vs. ferric sulfate; CEM vs. ferric sulfate	6–24 months	MTA vs. FS at 24 months: clinical RR = 0.98 (95% CI: 0.15–6.34; I^2^ = 0%); radiographic RR = 0.74 (0.23–2.43; I^2^ = 0%). Biodentine vs. FS at 6 months: clinical RR = 1.00 (0.95–1.05; I^2^ = 0%); radiographic RR = 0.99 (0.88–1.11; I^2^ = 51%). CEM vs. FS at 6 months: radiographic RR = 0.25 (0.03–2.22; I^2^ = 0%).
Lu et al. [27]	Biodentine vs. MTA	6, 12, 18, 24 months	Clinical RR: 1.00 (95% CI: 0.98–1.02), 1.01 (0.97–1.04), 0.99 (0.95–1.04), and 1.00 (0.94–1.07), respectively. Radiographic RR: 0.99 (0.97–1.02), 1.00 (0.96–1.03), 1.01 (0.94–1.09), and 1.01 (0.94–1.08), respectively. No significant differences.
Beldar et al. [28]	MTA and other calcium-silicate-based materials vs. conventional pulpotomy materials	≥12 months	Pooled MTA clinical success = 97.02%; pooled radiographic success = 94.21%. The review also reported material-specific comparative estimates for calcium-silicate-based and conventional pulpotomy materials using heterogeneous analytical approaches.

Effect estimates are reproduced at the systematic-review level as reported by the original meta-analyses and were not statistically recombined in the present umbrella review. Exact effect estimates are presented where they were numerically reported and could be directly extracted from the corresponding review. Shafaee et al. [8] analyzed failure rather than success; therefore, the direction of its effect estimates is not directly interchangeable with reviews reporting success. Bossù et al. [25] did not perform a meta-analysis and is therefore not represented in this table. RR, risk ratio; CI, confidence interval; TSA, trial sequential analysis; MTA, mineral trioxide aggregate; CEM, calcium-enriched mixture cement; FS, ferric sulfate.

**Table 4:** GRADE-informed certainty of evidence by material comparison and outcome.

Comparison	Outcome	Evidence Pattern	Main Reasons for Downgrading	Upgrading	Final Certainty
MTA vs. Biodentine	Clinical success	Broadly comparable outcomes across eight quantitative reviews through 24 months	Serious methodological limitations; substantial redundancy of primary evidence across reviews	None	Low
MTA vs. Biodentine	Radiographic success	Generally comparable; isolated findings favored MTA at selected follow-up intervals	Serious methodological limitations; inconsistency at selected follow-up intervals	None	Low
Biodentine vs. formocresol	Clinical success	No consistent clinically relevant difference	Serious methodological limitations; limited comparison-specific evidence/precision	None	Low
Biodentine vs. formocresol	Radiographic success	Inconsistent across reviews	Methodological limitations; inconsistency across reviews; limited independent evidence	None	Low
Biodentine vs. ferric sulfate	Clinical/radiographic success	No consistent significant difference	Serious methodological limitations; limited comparison-specific evidence	None	Low
MTA vs. ferric sulfate	Clinical/radiographic success	Inconsistent review-level findings; no significant difference in one meta-analysis; whereas, another favored MTA	Serious methodological limitations; inconsistency and serious imprecision	None	Very low
CEM vs. ferric sulfate	Radiographic success	No significant difference; sparse evidence	Serious methodological limitations; very serious imprecision/sparsity	None	Very low
MTA vs. Portland cement	Clinical/radiographic success	No consistent significant difference	Methodological limitations; serious imprecision because evidence was derived primarily from one review and few direct comparisons	None	Low
MTA vs. TheraCal	Clinical/radiographic success	No consistent significant difference	Methodological limitations; serious imprecision because evidence was derived primarily from one review and few direct comparisons	None	Low
First- vs. second-generation bioceramics	Clinical/radiographic success	Broadly comparable outcomes	Serious methodological limitations; serious imprecision	None	Low

Certainty judgments were made at the comparison–outcome level using a GRADE-informed approach adapted for umbrella reviews. Randomized-trial-dominant evidence initially received a high-certainty rating; whereas, evidence materially informed by non-randomized studies started at moderate certainty. One-level and two-level downgrades corresponded to serious and very serious concerns, respectively. Primary-study overlap was considered when repeated findings across systematic reviews did not represent independent replication and was not double-counted when its consequences were already reflected in another domain. No outcome fulfilled the predefined criteria for upgrading. AMSTAR-2, A Measurement Tool to Assess Systematic Reviews 2; CEM, calcium-enriched mixture cement; MTA, mineral trioxide aggregate.

## Data Availability

The datasets used and/or analyzed during the current study are available from the corresponding author upon reasonable request.

## Supplementary Materials

The following supporting information can be downloaded at: https://www.mdpi.com/article/10.3390/jcm15166379/s1, Table S1. Search strategies used for database searches. Table S2. Complete 16-item AMSTAR-2 assessment of the systematic reviews included in the umbrella review. Table S3. Systematic reviews excluded after full-text assessment. Table S4. Overlap matrix of primary-study reports across the 11 systematic reviews included in the umbrella review; File S1 PRIOR Reporting Checklist; PRISMA 2020 Abstract Checklist; PRISMA 2020.
