# Supplementary material for: Clinical and Radiographic Outcomes of Calcium-Silicate-Based Materials in Primary-Tooth Pulpotomy: An Umbrella Review
Source: J Clin Med. 2026 Aug 18;15(16):6379. doi: 10.3390/jcm15166379 (PMC13513962; doi:10.3390/jcm15166379)
Supplement: Supplementary file 1 [file jcm-15-06379-s001.zip › PRISMA 2020 Abstracts Checklist.pdf]

PRISMA 2020 for Abstracts Checklist

Clinical and Radiographic Outcomes of Calcium-Silicate-Based Materials in Primary-Tooth Pulpotomy: An Umbrella Review

Section	Topic	Item	PRISMA 2020 for Abstracts checklist item	Reported	Location in abstract / clarification
TITLE	Title	1	Identify the report as a systematic review.	Yes*	Title: identified as "An Umbrella Review."
BACKGROUND	Objectives	2	Provide an explicit statement of the main objective(s) or question(s) the review addresses.	Yes	Background/Objectives: main objective explicitly stated.
METHODS	Eligibility criteria	3	Specify the inclusion and exclusion criteria for the review.	Yes	Methods: eligible reports were systematic reviews of human primary-tooth pulpotomy involving calcium-silicate-based materials and reporting clinical and/or radiographic outcomes.
METHODS	Information sources	4	Specify the information sources (e.g. databases, registers) used to identify studies and the date when each was last searched.	Yes	Methods: MEDLINE/PubMed, Embase, and Scopus; searched from inception through June 2026.
METHODS	Risk of bias	5	Specify the methods used to assess risk of bias in the included studies.	Yes*	Methods: methodological quality of the included systematic reviews assessed with AMSTAR-2; adapted to the umbrella-review level.
METHODS	Synthesis of results	6	Specify the methods used to present and synthesise results.	Yes	Methods: structured narrative synthesis and review-level functional meta-synthesis; overlap assessed with CCA and certainty with a GRADE-informed approach.
RESULTS	Included studies	7	Give the total number of included studies and participants and summarise relevant characteristics of studies.	Yes*	Results: 11 systematic reviews, 78 unique primary-study reports, and 180 study occurrences. Aggregate participant totals were not calculated because primary studies recur across reviews.
RESULTS	Synthesis of results	8	Present results for main outcomes, preferably indicating the number of included studies and participants for each. If meta-analysis was done, report the summary estimate and confidence/credible interval. If comparing groups, indicate the direction of the effect (i.e. which group is favoured).	Yes	Results: main clinical/radiographic findings and direction of comparisons are reported; the umbrella review did not perform a de novo meta-analysis.
DISCUSSION	Limitations of evidence	9	Provide a brief summary of the limitations of the evidence included in the review (e.g. study risk of bias, inconsistency and imprecision).	Yes	Results/Conclusions: certainty was predominantly low; limitations included methodological weaknesses, high primary-study overlap, imprecision for sparsely studied comparisons, and incomplete reporting-bias assessment.
DISCUSSION	Interpretation	10	Provide a general interpretation of the results and important implications.	Yes	Conclusions: high success is interpreted cautiously; no material demonstrated sustained long-term superiority.
OTHER	Funding	11	Specify the primary source of funding for the review.	No	Funding is reported in the manuscript Funding statement but is not stated within the abstract.
OTHER	Registration	12	Provide the register name and registration number.	Yes	Methods: PROSPERO registration CRD420261338396.

*Adaptation for an umbrella review: the report is explicitly identified as an umbrella review, and AMSTAR-2 was used to appraise the included systematic reviews. Because primary studies recur across reviews (CCA 13.1%, high overlap), summing participants across reviews would risk double counting.

Source: PRISMA 2020 for Abstracts Checklist, from Page MJ, McKenzie JE, Bossuyt PM, et al. The PRISMA 2020 statement: an updated guideline for reporting systematic reviews. BMJ. 2021;372:n71. doi:10.1136/bmj.n71.
