# Supplementary material for: Clinical and Radiographic Outcomes of Calcium-Silicate-Based Materials in Primary-Tooth Pulpotomy: An Umbrella Review
Source: J Clin Med. 2026 Aug 18;15(16):6379. doi: 10.3390/jcm15166379 (PMC13513962; doi:10.3390/jcm15166379)
Supplement: Supplementary file 1 [file jcm-15-06379-s001.zip › PRISMA 2020.pdf]

PRISMA 2020 Checklist

Section and Topic	Item #	Checklist item	Location where item is reported
**Clinical and Radiographic Outcomes of Calcium-Silicate-Based Materials in Primary-Tooth Pulpotomy: An Umbrella Review**			
Title	1	Identify the report as a systematic review.	Title.
**ABSTRACT**			
Abstract	2	See the PRISMA 2020 for Abstracts checklist.	Abstract; see the PRISMA 2020 for Abstracts checklist.
**INTRODUCTION**			
Rationale	3	Describe the rationale for the review in the context of existing knowledge.	Section 1, Introduction.
Objectives	4	Provide an explicit statement of the objective(s) or question(s) the review addresses.	Section 1, final paragraph; Section 2.2 includes the explicit PICO question.
**METHODS**			
Eligibility criteria	5	Specify the inclusion and exclusion criteria for the review and how studies were grouped for the syntheses.	Section 2.2, Eligibility criteria.
Information sources	6	Specify all databases, registers, websites, organisations, reference lists and other sources searched or consulted to identify studies. Specify the date when each source was last searched or consulted.	Section 2.3, Information sources and search strategy.
Search strategy	7	Present the full search strategies for all databases, registers and websites, including any filters and limits used.	Section 2.3 and Supplementary Table S1.
Selection process	8	Specify the methods used to decide whether a study met the inclusion criteria of the review, including how many reviewers screened each record and each report retrieved, whether they worked independently, and if applicable, details of automation tools used in the process.	Section 2.4, Study selection.
Data collection process	9	Specify the methods used to collect data from reports, including how many reviewers collected data from each report, whether they worked independently, any processes for obtaining or confirming data from study investigators, and if applicable, details of automation tools used in the process.	Section 2.5, Data extraction.
Data items	10a	List and define all outcomes for which data were sought. Specify whether all results that were compatible with each outcome domain in each study were sought (e.g. for all measures, time points, analyses), and if not, the methods used to decide which results to collect.	Sections 2.2 and 2.5.
Data items	10b	List and define all other variables for which data were sought (e.g. participant and intervention characteristics, funding sources). Describe any assumptions made about any missing or unclear information.	Section 2.5; methodological clarification by author contact is described in Section 2.3.
Study risk of bias assessment	11	Specify the methods used to assess risk of bias in the included studies, including details of the tool(s) used, how many reviewers assessed each study and whether they worked independently, and if applicable, details of automation tools used in the process.	Section 2.6 and Supplementary Table S2 (AMSTAR-2 appraisal of included systematic reviews).
Effect measures	12	Specify for each outcome the effect measure(s) (e.g. risk ratio, mean difference) used in the synthesis or presentation of results.	Sections 2.5 and 2.9. Effect estimates were extracted as reported by the included reviews; no de novo effect measure was specified for a second-order meta-analysis.
Synthesis methods	13a	Describe the processes used to decide which studies were eligible for each synthesis (e.g. tabulating the study intervention characteristics and comparing against the planned groups for each synthesis (item #5)).	Section 2.9. Eligibility for each review-level synthesis was organized by material/comparator, outcome, and follow-up.
Synthesis methods	13b	Describe any methods required to prepare the data for presentation or synthesis, such as handling of missing summary statistics, or data conversions.	Section 2.9. No de novo transformation of primary-study data was performed.
Synthesis methods	13c	Describe any methods used to tabulate or visually display results of individual studies and syntheses.	Sections 2.9 and 3.5; review-level findings are tabulated in Table 3 and summarized in Figures 2–3.
Synthesis methods	13d	Describe any methods used to synthesize results and provide a rationale for the choice(s). If meta-analysis was performed, describe the model(s), method(s) to identify the presence and extent of statistical heterogeneity, and software package(s) used.	Section 2.9. Structured narrative synthesis and review-level functional meta-synthesis were used; no second-order meta-analysis was performed.
Synthesis methods	13e	Describe any methods used to explore possible causes of heterogeneity among study results (e.g. subgroup analysis, meta-regression).	Section 2.9. Differences were explored at review level by material/comparator, outcome, follow-up interval, and direction of effect; no de novo meta-regression was performed.
Synthesis methods	13f	Describe any sensitivity analyses conducted to assess robustness of the synthesized results.	Not applicable: no de novo sensitivity analysis was performed.
Reporting bias assessment	14	Describe any methods used to assess risk of bias due to missing results in a synthesis (arising from reporting biases).	Sections 2.6 and 2.8. Reporting/publication-bias information from included reviews informed AMSTAR-2 and certainty assessments.
Certainty assessment	15	Describe any methods used to assess certainty (or confidence) in the body of evidence for an outcome.	Section 2.8. GRADE-informed, comparison–outcome-specific certainty assessment.
**RESULTS**			
Study selection	16a	Describe the results of the search and selection process, from the number of records identified in the search to the number of studies included in the review, ideally using a flow diagram.	Section 3.1 and Figure 1.
Study selection	16b	Cite studies that might appear to meet the inclusion criteria, but which were excluded, and explain why they were excluded.	Section 3.1 and Supplementary Table S3.
Study characteristics	17	Cite each included study and present its characteristics.	Section 3.2 and Table 1.
Risk of bias in studies	18	Present assessments of risk of bias for each included study.	Section 3.3, Table 2, and Supplementary Table S2. Primary-study risk of bias was not reassessed de novo.

Section and Topic	Item #	Checklist item	Location where item is reported
Results of individual studies	19	For all outcomes, present, for each study: (a) summary statistics for each group (where appropriate) and (b) an effect estimate and its precision (e.g. confidence/credible interval), ideally using structured tables or plots.	Section 3.5 and Table 3. Review-level pooled estimates are presented as reported by the included reviews; no de novo primary-study synthesis was performed.
Results of syntheses	20a	For each synthesis, briefly summarise the characteristics and risk of bias among contributing studies.	Sections 3.2–3.3 and Tables 1–2.
Results of syntheses	20b	Present results of all statistical syntheses conducted. If meta-analysis was done, present for each the summary estimate and its precision (e.g. confidence/credible interval) and measures of statistical heterogeneity. If comparing groups, describe the direction of the effect.	Section 3.5 and Table 3. No second-order meta-analysis was performed.
Results of syntheses	20c	Present results of all investigations of possible causes of heterogeneity among study results.	Section 3.6. Review-level discrepancies were examined by comparison, outcome, and follow-up; no de novo statistical heterogeneity analysis was performed.
Results of syntheses	20d	Present results of all sensitivity analyses conducted to assess the robustness of the synthesized results.	Not applicable: no de novo sensitivity analysis was performed.
Reporting biases	21	Present assessments of risk of bias due to missing results (arising from reporting biases) for each synthesis assessed.	Sections 3.3 and 3.8; Supplementary Table S2. No de novo publication-bias analysis was performed.
Certainty of evidence	22	Present assessments of certainty (or confidence) in the body of evidence for each outcome assessed.	Section 3.8 and Table 4.
**DISCUSSION**			
Discussion	23a	Provide a general interpretation of the results in the context of other evidence.	Section 4, Discussion.
Discussion	23b	Discuss any limitations of the evidence included in the review.	Section 4, Discussion (limitations of the evidence).
Discussion	23c	Discuss any limitations of the review processes used.	Section 4, Discussion (limitations of the umbrella-review evidence and methods).
Discussion	23d	Discuss implications of the results for practice, policy, and future research.	Section 4, Discussion (clinical implications and future research).
**OTHER INFORMATION**			
Registration and protocol	24a	Provide registration information for the review, including register name and registration number, or state that the review was not registered.	Abstract and Section 2.1: PROSPERO CRD420261338396.
Registration and protocol	24b	Indicate where the review protocol can be accessed, or state that a protocol was not prepared.	Section 2.1: PROSPERO registration CRD420261338396.
Registration and protocol	24c	Describe and explain any amendments to information provided at registration or in the protocol.	Sections 2.1–2.2: registered eligibility criteria, outcomes, information sources, and analytical framework were retained; application of eligibility at the systematic-review level was clarified.
Support	25	Describe sources of financial or non-financial support for the review, and the role of the funders or sponsors in the review.	Funding statement: no external funding.
Competing interests	26	Declare any competing interests of review authors.	Conflicts of Interest statement.
Availability of data, code and other materials	27	Report which of the following are publicly available and where they can be found: template data collection forms; data extracted from included studies; data used for all analyses; analytic code; any other materials used in the review.	Data Availability Statement and Supplementary Materials.

Note. PRISMA 2020 was used as complementary reporting guidance for general systematic-review elements. PRIOR was the primary overview-specific reporting guideline. PRISMA items referring to primary-study risk of bias, effect measures, and statistical syntheses were adapted to the systematic-review level because no de novo meta-analysis of primary studies was performed.

Source: Page MJ, McKenzie JE, Bossuyt PM, Boutron I, Hoffmann TC, Mulrow CD, et al. The PRISMA 2020 statement: an updated guideline for reporting systematic reviews. BMJ. 2021;372:n71. doi:10.1136/bmj.n71.
