# Supplementary material for: Clinical and Radiographic Outcomes of Calcium-Silicate-Based Materials in Primary-Tooth Pulpotomy: An Umbrella Review
Source: J Clin Med. 2026 Aug 18;15(16):6379. doi: 10.3390/jcm15166379 (PMC13513962; doi:10.3390/jcm15166379)
Supplement: Supplementary file 1 [file jcm-15-06379-s001.zip › Supplementary File S1 PRIOR Reporting Checklist.pdf]

Supplementary File S1. Completed PRIOR Reporting Checklist

Clinical and Radiographic Outcomes of Calcium-Silicate-Based Materials in Primary-Tooth Pulpotomy: An Umbrella Review

Completed author checklist based on the Preferred Reporting Items for Overviews of Reviews (PRIOR) framework. The reporting elements below are concise paraphrases; item numbering follows the PRIOR statement.

Section	Item	PRIOR reporting element (concise paraphrase)	Location in manuscript
TITLE	1	Identify the report as an overview of reviews.	Title: identified as an umbrella review.
ABSTRACT	2	Summarize the purpose, methods, and principal results.	Abstract.
INTRODUCTION	3	Explain the rationale in the context of existing knowledge.	Introduction, rationale paragraph preceding the objectives.
INTRODUCTION	4	State the objective(s) or review question(s).	Introduction, final paragraph; Section 2.2 includes the explicit PICO question.
METHODS	5a	Specify inclusion/exclusion criteria and whether supplemental primary studies were included.	Section 2.2. No supplemental primary studies were included as independent evidence units.
METHODS	5b	Specify the operational definition of a systematic review used for eligibility.	Section 2.2, Study design eligibility criteria.
METHODS	6	List all information sources and final search/consultation dates.	Section 2.3. MEDLINE/PubMed, Embase, Scopus, reference-list screening and forward citation searches; final database update June 2026.
METHODS	7	Provide reproducible full search strategies and report limits/filters.	Section 2.3 and Supplementary Table S1.
METHODS	8a	Describe study-selection methods and reviewer roles.	Section 2.4. AMVB and EPV screened independently; CMA resolved unresolved disagreements.
METHODS	8b	Describe how overlap across reviews was identified/managed during selection.	Sections 2.4 and 2.7. All otherwise eligible reviews were retained; overlap was formally evaluated using a citation matrix and CCA.
METHODS	9a	Describe data-collection methods and reviewer roles.	Section 2.5. AMVB and EPV extracted data independently; CMA was consulted when necessary.
METHODS	9b	Describe methods used to identify/manage primary-study overlap during data collection/synthesis.	Sections 2.7 and 2.9. Primary studies were mapped across reviews and CCA was used to quantify overlap.
METHODS	9c	Describe management of discrepant review-level data.	Section 2.9. Differences were retained by material comparison, outcome type, follow-up interval and direction of effect.
METHODS	10	List variables/outcomes sought and handling of missing/unclear information.	Sections 2.2 and 2.5; author contact for methodological clarification is reported in Section 2.3.
METHODS	11a	Describe appraisal of methodological quality/risk of bias of included systematic reviews.	Section 2.6 and Supplementary Table S2 (AMSTAR-2).
METHODS	11b	Describe collection or reassessment of primary-study risk-of-bias information.	Section 2.8. Risk-of-bias information reported by contributing reviews informed the GRADE-based certainty assessment; no de novo primary-study reassessment was performed.
METHODS	11c	Describe risk-of-bias assessment for supplemental primary studies, if included.	Not applicable: no supplemental primary studies were included as independent evidence units.
METHODS	12a	Describe and justify methods used to summarize/synthesize results.	Section 2.9. Structured narrative synthesis and review-level functional meta-synthesis; no second-order statistical pooling.
METHODS	12b	Describe methods used to explore heterogeneity or differences in findings.	Section 2.9. Findings were examined across material/comparator, outcome, follow-up interval and direction of effect.
METHODS	12c	Describe sensitivity analyses, if performed.	Not applicable: no de novo meta-analysis or de novo sensitivity analysis was performed.
METHODS	13	Describe methods for assessing or collecting information on reporting bias.	Sections 2.6 and 2.8. Reporting/publication-bias information from included reviews was considered in AMSTAR-2 and certainty assessment.
METHODS	14	Describe certainty/confidence assessment methods.	Section 2.8. Comparison-outcome-specific GRADE-informed assessment with explicit downgrading/upgrading criteria.
RESULTS	15a	Report search and selection results, ideally with a flow diagram.	Section 3.1 and Figure 1.
RESULTS	15b	List full-text reports excluded after assessment and give reasons.	Supplementary Table S3.
RESULTS	16	Cite included reviews and present their characteristics.	Section 3.2 and Table 1.
RESULTS	17	Describe the extent of primary-study overlap.	Section 3.4 and Supplementary Table S4: 78 unique primary-study reports, 180 study occurrences, and CCA 13.1%, indicating high overlap.
RESULTS	18a	Present methodological-quality/risk-of-bias assessments for each included systematic review.	Section 3.3, Table 2 and Supplementary Table S2.
RESULTS	18b	Present primary-study risk-of-bias information collected or reassessed.	Sections 3.3 and 3.8. Primary-study risk-of-bias information was considered as reported by contributing reviews; no de novo primary-study assessment was performed.
RESULTS	18c	Present risk-of-bias assessments for supplemental primary studies, if included.	Not applicable: no supplemental primary studies were included as independent evidence units.
RESULTS	19a	Summarize evidence for all outcomes, including effect direction and relevant estimates/precision.	Sections 3.5–3.7, Table 3, and Figures 2–3.
RESULTS	19b	Present investigations of heterogeneity from any de novo meta-analysis.	Not applicable: no de novo meta-analysis was performed.
RESULTS	19c	Present sensitivity analyses from any de novo meta-analysis.	Not applicable: no de novo meta-analysis was performed.

Section	Item	PRIOR reporting element (concise paraphrase)	Location in manuscript
RESULTS	20	Present reporting-bias assessments where available.	Sections 3.3 and 3.8. Reporting/publication-bias assessments from the included reviews were considered in the AMSTAR-2 and certainty assessments; no de novo publication-bias analysis was performed.
RESULTS	21	Present certainty/confidence assessments for relevant outcomes.	Section 3.8 and Table 4.
DISCUSSION	22a	Summarize main findings and discrepancies across reviews.	Discussion.
DISCUSSION	22b	Interpret findings in the context of other evidence.	Discussion.
DISCUSSION	22c	Discuss limitations of the included evidence and overview methods.	Discussion, strengths and limitations paragraph.
DISCUSSION	22d	Discuss implications for practice/policy/future research and relevance to end users.	Discussion.
OTHER INFORMATION	23a	Provide registration information.	Abstract and Section 2.1: PROSPERO CRD420261338396.
OTHER INFORMATION	23b	State where the protocol can be accessed.	Section 2.1: PROSPERO database, registration CRD420261338396.
OTHER INFORMATION	23c	Describe and explain amendments/deviations from registration/protocol.	Sections 2.1–2.2: the registered eligibility criteria, outcomes, information sources, and analytical framework were retained; application of the eligibility criteria at the systematic-review level was clarified.
OTHER INFORMATION	24	Describe financial/non-financial support and funder role.	Funding statement: no external funding.
OTHER INFORMATION	25	Declare competing interests.	Conflicts of Interest statement.
OTHER INFORMATION	26a	Provide corresponding-author contact information.	Title page.
OTHER INFORMATION	26b	Describe author contributions and identify the guarantor.	Author Contributions statement; corresponding author identified on title page.
OTHER INFORMATION	27	State availability of data, forms, code and other materials.	Data Availability Statement and Supplementary Materials.

Reference: Gates M, Gates A, Pieper D, et al. Reporting guideline for overviews of reviews of healthcare interventions: development of the PRIOR statement. BMJ. 2022;378:e070849. doi:10.1136/bmj-2022-070849.
