# Supplementary material for: Clinical and Radiographic Outcomes of Calcium-Silicate-Based Materials in Primary-Tooth Pulpotomy: An Umbrella Review
Source: J Clin Med. 2026 Aug 18;15(16):6379. doi: 10.3390/jcm15166379 (PMC13513962; doi:10.3390/jcm15166379)
Supplement: Supplementary file 1 [file jcm-15-06379-s001.zip › Supplementary Table S1.pdf]

Supplementary Table S1. Search strategies used for database searches

Clinical and Radiographic Outcomes of Calcium-Silicate-Based Materials in Primary-Tooth Pulpotomy: An Umbrella Review

**Database**	**Search strategy**
PubMed / MEDLINE	("pulpotomy"[Title/Abstract] OR "vital pulp therapy"[Title/Abstract])<br>AND<br>("primary teeth"[Title/Abstract] OR "primary molar"[Title/Abstract] OR "deciduous teeth"[Title/Abstract])<br>AND<br>("bioceramic"[Title/Abstract] OR "calcium silicate"[Title/Abstract] OR "mineral trioxide aggregate"[Title/Abstract] OR MTA OR biodentine)<br>AND<br>("systematic review"[Publication Type] OR "systematic review"[Title/Abstract] OR "meta-analysis"[Title/Abstract])
Scopus	TITLE-ABS-KEY (pulpotomy OR "vital pulp therapy")<br>AND<br>TITLE-ABS-KEY ("primary teeth" OR "primary molar" OR "deciduous teeth")<br>AND<br>TITLE-ABS-KEY (bioceramic OR "calcium silicate" OR "mineral trioxide aggregate" OR MTA OR biodentine)<br>AND<br>TITLE-ABS-KEY ("systematic review" OR "meta-analysis")
Embase	('pulpotomy'/exp OR pulpotomy:ti,ab OR 'vital pulp therapy':ti,ab)<br>AND<br>('primary tooth'/exp OR 'deciduous tooth':ti,ab OR 'primary molar':ti,ab)<br>AND<br>('bioceramic':ti,ab OR 'calcium silicate':ti,ab OR 'mineral trioxide aggregate':ti,ab OR biodentine:ti,ab)<br>AND<br>('systematic review'/exp OR 'meta analysis'/exp OR 'systematic review':ti,ab OR 'meta-analysis':ti,ab)

Note. No publication-date or language restrictions were applied. The final search update was completed in June 2026.
