# Supplementary material for: Clinical and Radiographic Outcomes of Calcium-Silicate-Based Materials in Primary-Tooth Pulpotomy: An Umbrella Review
Source: J Clin Med. 2026 Aug 18;15(16):6379. doi: 10.3390/jcm15166379 (PMC13513962; doi:10.3390/jcm15166379)
Supplement: Supplementary file 1 [file jcm-15-06379-s001.zip › Supplementary Table S2.pdf]

Supplementary Table S2. Complete 16-item AMSTAR-2 assessment of the systematic reviews included in the umbrella review

Clinical and Radiographic Outcomes of Calcium-Silicate-Based Materials in Primary-Tooth Pulpotomy: An Umbrella Review

Panel A. AMSTAR-2 items 1–8

Review	1	2†	3	4†	5	6	7†	8
Stringhini Junior et al. [7]	Y	**N**	**N**	PY	Y	Y	**N**	Y
Shafaee et al. [8]	Y	**N**	**N**	PY	Y	Y	Y	Y
Firoozi et al. [9]	Y	PY	**N**	PY	Y	Y	**N**	PY
Silva et al. [10]	Y	PY	**N**	PY	Y	Y	PY	Y
Albernaz Neves et al. [11]	Y	PY	Y	PY	Y	Y	Y	Y
Xavier et al. [23]	Y	PY	**N**	PY	**N**	**N**	Y	Y
Nagendrababu et al. [24]	Y	PY	**N**	PY	Y	Y	Y‡	Y
Bossù et al. [25]	Y	**N**	**N**	**N**	Y	**N**	Y	Y
Gopinath et al. [26]	Y	PY	**N**	PY	Y	Y	Y	Y
Lu et al. [27]	Y	**N**	Y	PY	Y	Y	**N**	Y
Beldar et al. [28]	Y	PY	**N**	PY	**N**	**N**	**N**	PY

Codes: Y = Yes; PY = Partial Yes; N = No; NA = Not applicable. † Critical AMSTAR-2 domain.

Panel B. AMSTAR-2 items 9–16 and overall confidence

Review	9†	10	11†	12	13†	14	15†	16	Overall confidence
Stringhini Junior et al. [7]	Y	**N**	**N**	**N**	Y	Y	**N**	**N**	**Critically low**
Shafaee et al. [8]	Y	**N**	Y	**N**	Y	Y	Y	Y	**Low**
Firoozi et al. [9]	Y	**N**	Y	**N**	Y	Y	Y	Y	**Low**
Silva et al. [10]	Y	**N**	Y	**N**	Y	Y	Y	**N**	**Moderate**
Albernaz Neves et al. [11]	Y	Y	Y	**N**	Y	Y	**N**	**N**	**Low**
Xavier et al. [23]	PY	**N**	Y	**N**	Y	Y	**N**	Y	**Low**
Nagendrababu et al. [24]	Y	**N**	Y	Y	Y	Y	Y	Y	**Moderate**
Bossù et al. [25]	Y	**N**	**NA**	**NA**	Y	Y	**NA**	Y	**Critically low**
Gopinath et al. [26]	Y	**N**	Y	**N**	Y	Y	**N**	Y	**Low**
Lu et al. [27]	Y	**N**	Y	**N**	Y	Y	Y	Y	**Critically low**
Beldar et al. [28]	Y	**N**	**N**	**N**	**N**	**N**	**N**	**N**	**Critically low**

‡ Nagendrababu et al. [24]: no studies were excluded after full-text assessment; all eight full-text reports were included.

For Bossù et al. [25], items 11, 12, and 15 were rated NA because no meta-analysis was undertaken; the review used descriptive synthesis because of substantial clinical and methodological heterogeneity.

Overall confidence ratings were determined from the number and nature of critical and non-critical weaknesses and were not based on a numerical score.

Item-level judgments were assigned after examination of the complete full-text publication and all available supplementary materials for each systematic review. An item was rated “No” when the required methodological information could not be identified in these sources or when the reported methods did not satisfy the corresponding AMSTAR-2 criterion; “Partial Yes” was used when the criterion was only partially fulfilled.

A PY judgment for protocol registration indicates that registration was reported, but the full prespecified protocol and any deviations from it could not be completely verified from the material assessed. A PY judgment for the literature search indicates a broad search with reported databases, terms and/or dates, but not all supplementary AMSTAR-2 search elements were documented.

For Stringhini Junior et al. [7], item 11 was rated N because the statistical-combination methods were incompletely specified in the report despite presentation of pooled risk ratios; this conservative item-level judgment does not change the overall critically low confidence rating.

AMSTAR-2 item definitions used in this table

Item	Concise appraisal criterion	AMSTAR-2 domain
**1**	PICO components included in the research question and eligibility criteria	Non-critical
**2†**	Protocol established before conduct of the review and deviations adequately justified	Critical
**3**	Selection of eligible study designs explained	Non-critical
**4†**	Comprehensive literature search strategy	Critical
**5**	Study selection performed in duplicate	Non-critical
**6**	Data extraction performed in duplicate	Non-critical
**7†**	Full-text exclusions listed and justified at the study level	Critical
**8**	Included studies described in adequate detail	Non-critical
**9†**	Satisfactory technique used to assess risk of bias in primary studies	Critical
**10**	Funding sources of included primary studies reported	Non-critical
**11†**	Appropriate methods used for statistical combination of results	Critical
**12**	Potential impact of primary-study risk of bias assessed in the meta-analysis or synthesis	Non-critical
**13†**	Primary-study risk of bias considered when interpreting or discussing review findings	Critical
**14**	Heterogeneity satisfactorily investigated, explained, or discussed	Non-critical
**15†**	Publication bias investigated and its likely impact discussed when quantitative synthesis permitted	Critical
**16**	Funding and potential conflicts of interest for the systematic review reported	Non-critical

Abbreviations: AMSTAR-2, A Measurement Tool to Assess Systematic Reviews 2; PICO, population, intervention, comparator, outcome.
