# Supplementary material for: Clinical and Radiographic Outcomes of Calcium-Silicate-Based Materials in Primary-Tooth Pulpotomy: An Umbrella Review
Source: J Clin Med. 2026 Aug 18;15(16):6379. doi: 10.3390/jcm15166379 (PMC13513962; doi:10.3390/jcm15166379)
Supplement: Supplementary file 1 [file jcm-15-06379-s001.zip › Supplementary Table S3.pdf]

Supplementary Table S3. Systematic reviews excluded after full-text assessment

Clinical and Radiographic Outcomes of Calcium-Silicate-Based Materials in Primary-Tooth Pulpotomy: An Umbrella Review

Study	Main focus of the review	Article-specific reason for exclusion
Coll et al., 2017	Comparative effectiveness of vital pulp therapy modalities in primary teeth, including indirect pulp treatment, direct pulp capping, and pulpotomy	The review-level question and synthesis were organized around the comparative effectiveness of different vital pulp therapy modalities rather than around calcium-silicate-based pulpotomy materials. Although MTA was evaluated within the pulpotomy component, the eligible material comparisons were embedded within a broader treatment-modality synthesis and did not constitute the organizing intervention of the systematic review.
Smaïl-Faugeron et al., 2018	Pulp treatment for extensive decay in primary teeth, including pulpotomy, pulpectomy, direct pulp capping, and multiple medicaments/techniques	The review evaluated multiple pulp-treatment modalities and medicaments for extensive decay in primary teeth. Although several pulpotomy analyses involved MTA, calcium-silicate-based materials represented only one component of a substantially broader pulp-treatment synthesis rather than the prespecified organizing intervention of the review.
Bastos et al., 2024	Long-term clinical effectiveness of Biodentine across vital pulp therapy procedures in primary and permanent teeth	The review evaluated Biodentine across multiple vital pulp therapy procedures and both dentitions. Although clinical and radiographic outcomes for primary-tooth pulpotomy were separately reported, the relevant review-level synthesis primarily pooled Biodentine success proportions rather than preserving a direct comparative material-versus-comparator synthesis specific to primary-tooth pulpotomy.
Lin et al., 2024	Outcomes of pulpotomy as a treatment modality for primary teeth diagnosed with irreversible pulpitis	The primary review question evaluated the effectiveness of pulpotomy as a treatment modality for irreversible pulpitis rather than the comparative performance of calcium-silicate-based pulpotomy materials. Material-specific comparison between MTA and non-MTA bioceramic materials was a secondary analysis and was based on only two of the five included primary studies.
Laser et al., 2024	Clinical and radiographic success of Biodentine and calcium hydroxide in primary-tooth pulpotomy	The review included non-controlled trials, prospective and retrospective cohort studies, and case series in addition to comparative trials. Its review-level synthesis pooled Biodentine and calcium-hydroxide success rates from separate study groups rather than being restricted to comparative clinical studies with eligible comparator groups, which was inconsistent with the predefined requirement for comparative evidence.

Note. MTA, mineral trioxide aggregate.
