# Supplementary material for: Clinical and Radiographic Outcomes of Calcium-Silicate-Based Materials in Primary-Tooth Pulpotomy: An Umbrella Review
Source: J Clin Med. 2026 Aug 18;15(16):6379. doi: 10.3390/jcm15166379 (PMC13513962; doi:10.3390/jcm15166379)
Supplement: Supplementary file 1 [file jcm-15-06379-s001.zip › Supplementary Table S4.pdf]

Supplementary Table S4. Overlap matrix of primary-study reports across the 11 systematic reviews included in the umbrella review

Clinical and Radiographic Outcomes of Calcium-Silicate-Based Materials in Primary-Tooth Pulpotomy: An Umbrella Review

Primary-study report	Stringhini [7]	Shafaee [8]	Firoozi [9]	Silva [10]	Albernaz Neves [11]	Xavier [23]	Nagendrababu [24]	Bossù [25]	Gopinath [26]	Lu [27]	Beldar [28]
Abd Al Gawad et al. 2021											✓
Abdelhafez et al. 2022				✓							
Agamy et al. 2004						✓		✓			✓
Ahuja et al. 2020			✓								
Alnassar et al. 2023						✓					✓
Alqahtani et al. 2023						✓					
Alsanouni et al. 2019					✓	✓					
Alzoubi et al. 2021				✓							
Ansari et al. 2010								✓			
Ansari et al. 2018									✓		
Baharloo et al. 2020			✓								
Bani et al. 2017	✓			✓	✓	✓	✓	✓		✓	✓
Carti et al. 2017	✓	✓		✓	✓			✓		✓	✓
Celik et al. 2013						✓		✓			
Chotitanmapong et al. 2019			✓								
Cuadros-Fernández et al. 2016	✓	✓		✓	✓		✓	✓		✓	✓
Doyle et al. 2010								✓	✓		
Eidelman et al. 2001								✓			
El Meligy et al. 2019		✓	✓					✓			
Elbardissy et al. 2019			✓								
Erdem et al. 2011								✓	✓		✓
Eshghi et al. 2022											✓
Farsi et al. 2005								✓			
Fernandez et al. 2013									✓		
Fouad et al. 2013	✓						✓				
Goyal et al. 2016									✓		
Guven et al. 2017		✓		✓	✓	✓		✓	✓		✓
Haghgoo et al. 2023											✓
Hassanpour et al. 2023				✓		✓					✓
Hidalgo et al. 2023				✓							
Holan et al. 2005											✓
Jamali et al. 2018								✓			
Jayam et al. 2014								✓			
Joo et al. 2023						✓					
Juneja et al. 2017	✓	✓	✓	✓			✓	✓		✓	✓
Junqueira et al. 2018								✓	✓		
Kamboj et al. 2019				✓							
Kang et al. 2015					✓	✓					✓
Khatab 2019			✓								
Khorakian et al. 2014											✓
Kiranmayi et al. 2022				✓							
Kusum et al. 2015	✓	✓		✓	✓	✓	✓			✓	
Lima et al. 2020				✓	✓	✓					
Liu et al. 2011											✓
Malekafzali et al. 2011					✓	✓		✓			✓
Manohar et al. 2022				✓							
Mazhari et al. 2017				✓							
Meslmani et al. 2020				✓							
Mettlach et al. 2013								✓			
Moretti et al. 2008								✓			✓
Niranjani et al. 2015	✓	✓		✓			✓			✓	

Primary-study report	Stringhini [7]	Shafaee [8]	Firoozi [9]	Silva [10]	Albernaz Neves [11]	Xavier [23]	Nagendrababu [24]	Bossù [25]	Gopinath [26]	Lu [27]	Beldar [28]
Noorollahian et al. 2008								✓			✓
Olatosi et al. 2015								✓			✓
Oliveira et al. 2013				✓		✓		✓			
Oznurhan et al. 2020					✓						
Rajasekharan et al. 2017	✓	✓		✓	✓	✓	✓	✓		✓	✓
Ramanandvignesh et al. 2020				✓		✓					
Rasteh et al. 2023											✓
Raval et al. 2017									✓		
Rubanenko et al. 2019			✓								
Sajadi et al. 2021									✓		
Sakai et al. 2009				✓		✓		✓			✓
Silva et al. 2019								✓			✓
Sirohi et al. 2017		✓							✓		✓
Sonmez et al. 2008								✓	✓		
Spandana et al. 2023				✓							
Srinivasan et al. 2011								✓			
Subramaniam et al. 2009								✓			
Sunitha et al. 2017								✓			
Sushynski et al. 2012								✓			
Togaru et al. 2016	✓	✓		✓	✓		✓	✓		✓	
Tungjitphianpong et al. 2024										✓	
Verma et al. 2019			✓								
Vilella-Pastor et al. 2021				✓	✓	✓				✓	✓
Yildirim et al. 2016				✓		✓		✓			
Yilmaz et al. 2023											✓
Zhao et al. 2024											✓
Çelik et al. 2019				✓	✓	✓		✓		✓	✓
**No. of eligible primary-study reports represented per review**	**9**	**10**	**9**	**26**	**14**	**20**	**8**	**33**	**11**	**11**	**29**

Note. ✓ indicates that the primary-study report was represented in the corresponding systematic review. The unit of mapping was the identifiable primary-study report. Bibliographic variants referring to the same publication were reconciled, and exact duplicate citations were counted once. Distinct publications were retained as separate reports when the available review-level information did not permit unequivocal linkage to the same underlying trial. The matrix contained 78 unique identifiable primary-study reports (r), 180 total study occurrences (N), and 11 systematic reviews (c). CCA = (N − r)/[(r × c) − r] = 13.1%, indicating high overlap. For systematic reviews with a broader scope, only the primary-study reports contributing to the eligible primary-tooth pulpotomy evidence involving at least one calcium-silicate-based material were represented. Bossù et al. [25] included 41 papers overall; 33 contributed to the eligible calcium-silicate-based evidence and were entered in this matrix. Beldar et al. [28] reported 30 studies, but 29 distinct primary-study reports could be identified from its study table/reference list; a duplicated Haghgoo et al. citation was represented once. The Alnassar trial is shown under its final bibliographic publication year (2023). CCA, corrected covered area; MTA, mineral trioxide aggregate.

Review key: Stringhini Junior et al. [7]; Shafaee et al. [8]; Firoozi et al. [9]; Silva et al. [10]; Albernaz Neves et al. [11]; Xavier et al. [23]; Nagendrababu et al. [24]; Bossù et al. [25]; Gopinath et al. [26]; Lu et al. [27]; Beldar et al. [28].
